# Counter-stereotypical messaging and partisan cues: Moving the needle on vaccines in a polarized United States

**DOI:** 10.1126/sciadv.adg9434

**Published:** 2023-07-19

**Authors:** Bradley J. Larsen, Timothy J. Ryan, Steven Greene, Marc J. Hetherington, Rahsaan Maxwell, Steven Tadelis

**Affiliations:** ^1^ Washington University in St. Louis, St. Louis, MO, and NBER, Cambridge, MA.; ^2^ The University of North Carolina at Chapel Hill, Chapel Hill, NC.; ^3^ North Carolina State University, Raleigh, NC.; ^4^ New York University, New York City, NY.; ^5^ The University of California at Berkeley, Berkeley, CA, and NBER, Cambridge, MA.

## Abstract

This paper reports results from a large-scale randomized controlled trial assessing whether counter-stereotypical messaging and partisan cues can induce people to get COVID-19 vaccines. The study used a 27-s video compilation of Donald Trump’s comments about the vaccine from Fox News interviews and presented the video to millions of U.S. YouTube users through a $100,000 advertising campaign in October 2021. Results indicate that the number of vaccines increased in the average treated county by 103 (with a one-tailed *P* value of 0.097). Based on this average treatment effect and totaling across our 1014 treated counties, the total estimated effect was 104,036 vaccines.

## Teaser

A nationwide field experiment shows how to reduce COVID vaccine hesitancy using political messengers.

## INTRODUCTION

In June 2021, U.S. White House chief medical advisor Anthony Fauci warned that coronavirus disease 2019 (COVID-19) vaccination disparities could lead to the emergence of “two Americas,” where regions with higher vaccination rates would fare much better than those with lower rates (*1*). At the time, vaccination was one of the most important tools for combatting the horrors of the virus, and it was a public health priority to get as many people vaccinated as possible.

Geographic divides in vaccination rates were driven by several factors, including differences in race, ethnicity, income, urbanicity, education, and age, which were all associated with Americans’ decisions to get vaccinated. However, political partisanship was the deepest fault line and seen by many as the key to understanding vaccination divides (*2*). Republican political leaders often downplayed the danger of COVID-19 while simultaneously amplifying false claims about the dangers of the vaccine. As a result, vaccine hesitancy emerged disproportionately among Republicans. The Kaiser Family Foundation estimated that, among the 27% of American adults who remained unvaccinated in late October 2021, 60% were Republicans, far above their share in the electorate (*3*, *4*). Consistent with Fauci’s warning, the division carried life and death consequences. By early fall, counties that voted heavily for Donald Trump experienced COVID-related death rates nearly three times higher than counties that voted heavily for Joe Biden (*5*).

We posited that a remedy for this partisan divide might come from the same mechanism that created the disparity in the first place: partisan cues. Research shows that partisans form preferences by following cues from their party leaders (*6*, *7*, *8*), a regularity that has grown stronger as the parties have polarized over the last generation (*9*). We hypothesized that messages publicizing Donald Trump’s support for COVID-19 vaccines—support Trump did little to advertise after leaving the White House—might cue some of the vaccine-hesitant among his supporters to get vaccinated themselves.

**Fig. 1.**
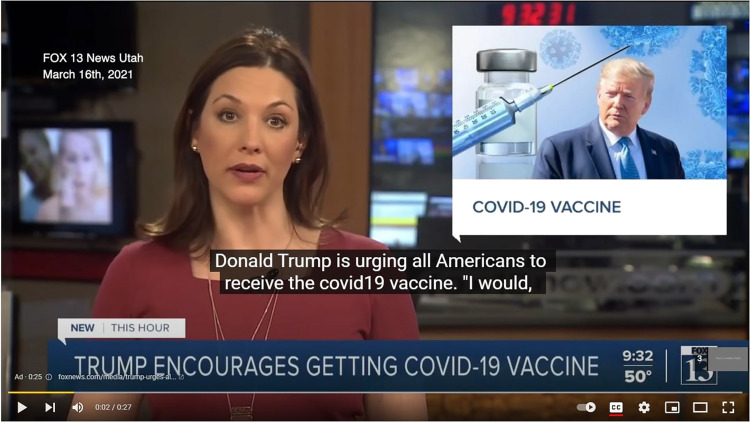
Screenshot of PSA at 3 s.

To test this hypothesis, we created a public service announcement (PSA), featuring news clips of Donald Trump on Fox News encouraging his supporters to get vaccinated. The PSA can be viewed at https://www.youtube.com/watch?v=INH-CmCgIYs; Fig. 1 shows a screenshot. By using both Trump and Fox—both of whom have long questioned the seriousness of the pandemic—we created a counter-stereotypical messenger, which theory predicts can be a particularly powerful catalyst (*10*).

We tested the PSA’s efficacy through a large randomized controlled trial (RCT) on YouTube, randomizing at the county level and targeting areas that lagged in vaccine uptake. Overall, we spent just under U.S. $100,000 in ads, with a total of 11.6 million ads reaching 6 million unique viewers. These ads appeared before YouTube viewers on mobile phones, TVs, tablets, and computers. Google’s algorithms attached our ad to specific YouTube channels and YouTube posts. The YouTube channel on which Google placed our ad the most was the Fox News channel (playing our ad over 200,000 times on this channel), with the ad displayed before segments of Sean Hannity, Laura Ingraham, Tucker Carlson, and other conservative cable news personalities. As such, our ad likely reached a disproportionately Republican audience, supporting our aim to offer a counter-stereotypical message.

We measure the effect of the campaign on county-level vaccination counts in Centers for Disease Control and Prevention (CDC) data. Previous work demonstrates that effects of ads on actual behavior tend to be miniscule and difficult to detect (*11*, *12*), challenges amplified by the fact that outcomes in our setting are measured at the county (rather than individual) level. Given the important potential live-saving implications of the study, we were both hopeful of moving the needle on vaccinations and circumspect about our ability to produce especially precise estimates of treatment effects given the study design.

Our intent-to-treat (ITT) analysis implies that the treatment led to an increase of 103 vaccinations in the average treated county, albeit with a fair amount of uncertainty (with a one-tailed *P* value of 0.097). We also exploit treatment intensity (the realized number of ad impressions) across treatment counties to estimate the average causal response (ACR) of actual ad displays. Using treatment assignment to instrument for the number of ads, we estimate an ACR of 1000 additional ads to be 8.6 vaccines per county. Alternatively, 116 impressions of our ad were required to move one additional viewer to get the vaccine. A one-standard-deviation increase in the number of ads per county results in 217 additional vaccines in the average county. We show that these results are robust to different ways of controlling for county population and to dropping counties with CDC data errors.

We also take advantage of the detailed engagement data provided by Google Ads tools. These data allow us to observe several alternative measures of treatment intensity in each county, such as how much it cost us (as bidding advertisers) to win the ad auctions corresponding to viewers in each county. Google’s tools also show, for each county, the percentage of viewers who watched at least 10 s of the ad, watched the full ad, or clicked on the ad’s link. In the average county, 41% of YouTube viewers to whom the ad was shown watched at least 10 s of the ad, although they were allowed to skip after 5 s, and 12% watched the full ad. We find that a one-standard-deviation increase in the percentage of viewers watching at least 10 s of the ad increases the number of vaccines distributed in the county by 8.2. A one-standard-deviation increase in the percentage of viewers watching the full ad leads to 12 additional vaccines.

We further expand our analysis by examining heterogeneous treatment effects. Among the Trump-leaning counties that comprise our sample, counties that are less heavily Trump-leaning were more responsive to the ad. Specifically, counties below the median level of Trump support (69.4%) saw treatment effects that were larger than counties with greater Trump support, and this difference is large enough and measured with sufficient precision that, in a two-tailed test, it is significant at the 0.10 level when vaccinations are measured in levels and at the 0.05 level when vaccinations are measured in rates.

With 1014 treated counties in all, our estimated treatment effect indicates that the total increase in vaccinations from the campaign was 104,036, resulting in about 1 vaccine per dollar in ad spending. Again, we emphasize that the treatment effect is less precisely estimated than would be ideal, but the results taken as a whole suggest that our approach likely made a difference. Combining these estimates with those of Barro (*13*) indicates that our campaign resulted in 839 avoided deaths, costing $115 per life saved. Given plentiful evidence of small, undetectable effects of public messaging in other settings, these results are encouraging and represent a large return on investment.

We join a growing body of literature on interventions to increase COVID-19 vaccinations, but our study is distinct in highlighting a political mechanism, and its success for what appears to be a fraction of the cost of other interventions. For example, Thirumurthy *et al*. (*14*) and Lang *et al*. (*15*) study state vaccine lotteries but fail to detect positive effects, while Barber and West (*16*) and Acharya and Dhakal (*17*) document positive effects for some states. An RCT in Sweden found an increase in COVID-19 vaccinations from a direct cash transfer, but at a much higher cost per vaccine ($24) (*18*). Chang *et al.* (*3*) run a similar RCT among Medicaid patients in the U.S. and find that even $50 was not enough to increase vaccinations. Dai *et al*. (*20*) study an intervention of text message reminders sent to U.S. adults to get the COVID-19 vaccine, and Bartoš *et al*. (*21*) study a campaign informing Czech citizens of doctors’ support for the vaccine. Both of those studies documented some positive effects. However, our study breaks new ground by leveraging partisan divides as a tool for improving public health. Partisan divides have long been the main divide in COVID-19 vaccination rates (*2*), but ours is the only academic study—to the best of our knowledge—to demonstrate that partisan divides can be leveraged as part of the solution.

## RESULTS

### Core results

The results of our ITT analysis are shown in columns 1 and 2 of Table 1. Throughout much of our analysis, we consider one-tailed tests for inference because our expectations are clearly directional: A campaign encouraging vaccination can only move the needle among the unvaccinated, who were predominantly Republican at this time (in October 2021, 91% of Democrats were vaccinated, compared to only 62% of Republicans) (*2*). In aspects of our analysis where we have no clear directional expectation (specifically, our analysis of heterogeneity in treatment effects), we apply two-tailed tests. See Materials and Methods for additional discussion of inference.

**Table 1.** Vaccine increase per county. Regression results. Sample size is 151,945 county-date observations. All regressions include fixed effects at the county and date levels. In columns 1 and 2, the ITT effect corresponds to the OLS-estimated coefficient on the interaction of a treatment assignment dummy (Treat) with a dummy for dates after October 14 (Post), the start of the campaign. Column 1 also includes the interaction of Post with county population. Column 2 replaces this with interactions of county population with (i) flexible dummies for each date within 2 weeks before to 2 weeks after the campaign (omitting the date before the campaign started), (ii) a dummy variable for 2 weeks or more before, and (iii) a dummy variable for 2 weeks or more after. Columns 3 and 4 report the IV-estimated coefficient on the interaction of the number of ads the county received (in 1000s) with Post, with this interaction instrumented for by Treat × Post. Column 3 mimics column 1 in controlling for differential trends by population, and column 4 mimics column 2. “***,” “**,” and “*” indicate significance (from a one-tailed test) at the 0.01, 0.05, and 0.10 levels. Standard errors, reported in parentheses below each estimate, are clustered at the county level. Randomization inference *P* values are from a one-tailed test based on 1000 permutations using the treatment effect as the randomization test statistic. Table S4 contains estimates of other coefficients from these regressions.

	ITT effect		ACR of 1000 ads	
	(1)	(2)	(3)	(4)
Effect	102.6* (78.74)	101.4* (78.76)	8.606* (6.608)	8.500* (6.609)
Implied vaccines per dollar	1.08* (0.828)	1.07* (0.828)	1.01* (0.773)	0.99* (0.773)
				
County fixed effects	Yes	Yes	Yes	Yes
Date fixed effects	Yes	Yes	Yes	Yes
Population × Post dummy	Yes		Yes	
Population × Date dummies		Yes		Yes
Randomization inference *P* value	0.067	0.065		

Each column in Table 1 reports effects from a difference-in-difference analysis, controlling for vaccine levels differing across counties and dates. Our effect of interest is the average treatment effect of the campaign for the average treatment county—the increase in vaccine first doses that would be expected by the average county if it were to adopt this campaign. Column 1 controls for differential trends by county population size through an interaction of county population with a dummy for the period after the start of the campaign. Column 2 includes more flexible controls for these trends that interact county population size with separate dummy variables for individual dates. The results are statistically indistinguishable across the two columns, demonstrating that the results are not sensitive to how we control for the growth rate of vaccinations in different sized counties. In column 1, we observe an increase of 102.6 vaccines, with a standard error of 78.74, implying a *P* value from a one-tailed test of 0.097. Thus, at a 95% confidence level, we cannot reject a null effect. At a 90% confidence level, we do reject zero, although we still cannot rule out small effects (as low as 1.81 vaccines per county).

As we discuss in Materials and Methods, these confidence bounds are constructed under our most conservative clustering approach (county-level clustering). We obtain smaller standard errors (and hence smaller *P* values) under every alternative level of clustering we apply, including state-level or stratum-level clustering. Given that we know the design for the field experiment, our study also lends itself particularly well to randomization inference. Throughout the study, we report *P* values using the treatment effect as our test statistic for randomization inference tests of the weak null hypothesis (the hypothesis that a treatment effect is zero for all counties). See Materials and Methods for more details. The randomization inference *P* value for column 1 is 0.067, again suggesting a significant effect at the 90% confidence level but not at the 95% level. Column 2 yields similar estimates and inference.

We discuss here implications of the point estimates, keeping in mind that, based on the standard errors, we cannot reject much smaller effects. The estimate in column 1 implies that, among the 1014 treatment counties, the average county saw a causal increase of 102.6 vaccines due to the campaign. Aggregating across all treatment counties, the total effect was an increase of 104,036 vaccines. Because our total ad budget spent in these counties was $96,408.56, the estimated vaccines per dollar spent was 1.08 (standard error of 0.828), with similar results in column 2. In either case, this implies that $1 in advertising spending yielded one more shot in the arm. It bears noting that, at the lower 90% confidence bound, the estimated effect is quite small: Only 0.017 vaccines resulted from $1 in ads, or $58.8 dollars per vaccine. At our 90% lower confidence bound, this is still more cost effective than the point estimates of $68 and $82 in *(**16*, *17*), respectively. At a 95% confidence level, we would be unable to reject much larger cost estimates.

Estimates from Barro (*13*) can be used for a back-of-the-envelope calculation to translate our results into an estimated number of lives saved. Barro (*13*) estimates that, in the latter part of 2021, 124 additional full vaccine sequences were sufficient to avoid one additional COVID-19 death. If each of the 104,036 first-dose vaccines from our campaign eventually yield fully vaccinated sequences—clearly a strong assumption—these estimates would imply that 839 deaths were avoided because of our campaign, costing $115 per life saved. Although we are hopeful that the campaign saved many lives, it is again important to note that the confidence intervals surrounding these estimates are wide.

Columns 3 and 4 of Table 1 use the level of ad exposure to identify the causal response of an additional 1000 ads in a county in an instrumental variables (IV) framework, instrumenting for the number of ads using treatment assignment. We again offer two different approaches to controlling for differential trends by county population, as in columns 1 and 2. Both columns 3 and 4 show a positive and significant (and similarly sized) effect. The estimate in column 3 of Table 1 suggests that an increase of 1000 ads leads to 8.6 additional vaccines in the average treated county. Put differently, 116 ad impressions (1000 divided by 8.6) are required to yield one additional vaccine. Because the standard deviation of the number of ads across treatment counties is 25,245 (from Table 1), the estimate in column 3 implies that a one-standard-deviation increase in ads yields 217 additional vaccines in the county.

As described in Materials and Methods, 1000 ad impressions cost us $8.55 on average (the cost per mille, or “CPM” in advertising lingo). These numbers therefore imply an alternative estimate of the number of vaccines per dollar, at 1.01. The estimate in column 4 implies a similar estimate of cost effectiveness, at 0.99 vaccines per dollar spent. Thus, regardless of whether we rely on the estimated ITT effect or the ACR, our point estimates imply that $1 in advertising spending persuaded one (or slightly more than one) viewer to get the vaccine. Supplementary Materials (SM) Section A presents various robustness checks on the results in this section.

So far, we have conceptualized our outcome measure as a level: the number of people in a county who are vaccinated. An alternative approach would be to conceptualize the dependent measure as a rate: the percent of residents in a county who are vaccinated. We report results from this analysis in Table 2. For the measure of ad exposure in columns 3 and 4, we use the number of ads per 100 residents in the county. Table 2A displays results from these regressions without weighting observations (as in Table 1), and Table 2B shows results where each observation is weighted by the size of the county.

**Table 2.** Measuring vaccines and ads in rates. This table displays estimates from modifications of Eqs. 1 and 2 where the dependent variable is the total percent of the county population vaccinated at a given point in time and the treatment intensity is measured as the number of ads a county receives per 100 residents. The number of observations is slightly higher here than in our main analysis (163,856 county-date observations rather than 151,945) because, for some observations, the vaccination count is missing on certain dates in the CDC data, although the vaccination rate is recorded. “***,” “**,” and “*” indicate significance (from a one-tailed test) at the 0.01, 0.05, and 0.10 levels. Standard errors, reported in parentheses below each estimate, are clustered at the county level. Randomization inference *P* values are from a one-tailed test based on 1000 permutations using the treatment effect as the randomization test statistic. Panel (**A**) reports results from an unweighted regression, and panel (**B**) shows results where observations are weighted by county population. Table S5 contains estimates of other coefficients from these regressions.

	ITT effect		ACR of 1000 ads	
	(1)	(2)	(3)	(4)
**A. Unweighted regression**				
Effect	0.570* (0.437)	0.563* (0.437)	0.0296* (0.0227)	0.0291* (0.0226)
Randomization inference *P* value	0.089	0.093	–	–
**B. Weighted regression**				
Effect	0.448 (0.458)	0.411 (0.454)	0.0191 (0.0196)	0.0173 (0.0192)
Randomization inference *P* value	0.159	0.184	–	–
County fixed effects	Yes	Yes	Yes	Yes
Date fixed effects	Yes	Yes	Yes	Yes

In Table 2A, we find point estimates that are positive and significant at the 0.10 level, under either county-level clustering or randomization inference. The estimates in column 1 suggest the campaign increased the percent vaccinated (a variable ranging from 0 to 100) by 0.57 percentage points in the average county. Similarly, the results in column 3 suggest that an increase of 1 more ad per 100 county residents increases the percent vaccinated by 0.03 percentage points. Comparing odd and even columns, we see very similar point estimates, suggesting that controlling directly for county population after normalizing by county population does little to affect the estimates. When we weight by county population in Table 2B, we find slightly smaller estimates, enough to yield results that are no longer significant at the 0.10 level, illustrating that the marginal statistical significance of our results is not robust to this variation in the estimation approach.

**Fig. 2.**
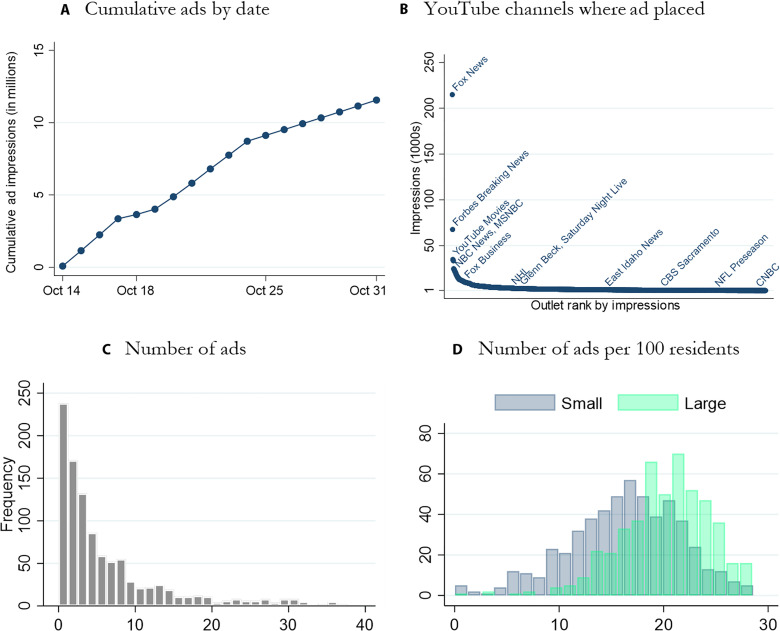
Ad campaign characteristics. (**A**) Cumulative ads by date (in millions). (**B**) Each dot represents a given YouTube channel on which the ad was shown over 1000 times (with only a handful of these channels labeled for readability). The vertical axis shows the number of times (in units of 1000) that the ad showed on a given channel, and the horizontal axis orders channels in descending order by the number of times the ad showed. (**C**) Histogram of the number of ads per county (in thousands). (**D**) Number of ads per 100 county residents, separately for small (below median population) and large (above median population) counties.

As demonstrated in the histograms in Fig. 2 (C and D) and in the surrounding discussion, the number of ad impressions varies widely across counties, but the number of ads per capita does as well. Hence, the decision of whether to measure ads in rates or levels has no single correct answer, as the actual meaning of being “treated” is not fully captured by either. We prefer the levels-focused analysis because we pre-registered it to address our primary research question of whether our ad campaign changed behavior sufficiently to increase the total count of vaccinated individuals (regardless of how large this change is relative to county size).

### Other measures of treatment intensity

The number of ads a county receives is only one of several ways to measure a county’s treatment intensity. Using the same IV framework as above, we examine several other measures that represent viewer engagement within a county, including the engagement rate, view rate, click rate, and CPM, as well as the frequency of ads relative to the county population (the number of ads per 100 residents). These variables are defined in more detail below. We normalize each variable by its standard deviation (across treatment counties) to facilitate comparison. Thus, each ACR can be interpreted as the effect of a one-standard-deviation increase in the corresponding variable. It is important to note that these measures of treatment intensity covary in complex ways (table S2). Our analysis here measures the ACR to these measures in separate regressions, but we emphasize that we are not capturing the causal effect of independently moving a single measure of treatment intensity.

The results are displayed in Table 3. We estimate that a one-standard-deviation increase in the percentage of viewers watching at least 10 s of the ad (engagement rate) increases vaccines distributed in the average county by 8.2. Additionally, a one-standard-deviation increase in the rate of those viewing the full ad (view rate) increases vaccinations by 12.3. Both suggest that counties in which viewers watched the ad for longer were the most responsive in terms of vaccinations. Other measures of viewer engagement produce even larger effects. A one-standard-deviation increase in the rate of clicking on the Fox News link at the bottom of the ad (click rate) leads to 94.1 additional vaccines. These results are also reflected in the cost of ads: A one-standard-deviation increase in a county’s CPM results in 4.9 additional vaccines, suggesting that, while we had to bid more for each ad in some counties than others, this increased spending yielded viewers who were more likely to respond by getting the vaccine. Finally, ad coverage affects uptake as well, with a one-standard-deviation increase in the number of ads per capita in the county resulting in 48.4 additional vaccines. All these effects are statistically significant at the 0.10 level regardless of how we control for population (panel A versus panel B) but not the 0.05 level. These results together suggest that as viewers engage more with the ad, the persuasive effects of the ad may be stronger.

**Table 3.** Vaccine increase per county based on engagement metrics. Regression results from IV regressions, as in columns 3 and 4 of Table 1, but using different treatment intensity measures instead of number of ads. The dependent variable is the same as in Table 1, but the measure of treatment intensity differs by column. Each variable is normalized by dividing by its standard deviation across counties. A county’s engagement rate is the number of 10-s (or longer) views of the ad, divided by the number of ad impressions the county received, multiplied by 100. View rate is the number of complete views of the ad divided by the number of impressions, multiplied by 100. Click rate is the number of times the Fox News story link was clicked, divided by the number of ads, multiplied by 100. CPM is the average cost (in dollars) of purchasing 1000 ad impressions in the county. Panels (A) and (B) control for differential trends over time in counties of different populations using the strategies in Table 1; panel (A) follows column 3 of Table 1 and panel (B) follows column 4 of Table 1. “***,” “**,” and “*” indicate significance (from a one-tailed test) at the 0.01, 0.05, and 0.10 levels. Standard errors, reported in parentheses below each estimate, are clustered at the county level.

Treatment intensity measure	Engagement rate	View rate	Click rate	Ads per 100 residents	CPM
	(1)	(2)	(3)	(4)	(5)
**A. Controlling for population × Post dummy**					
Average causal response	8.255* (6.333)	12.34* (9.467)	94.12* (72.11)	48.37* (37.10)	4.877* (3.742)
**B. Controlling for population × Date dummies**					
Average causal response	8.153* (6.335)	12.19* (9.469)	92.96* (72.12)	47.77* (37.10)	4.817* (3.742)
County fixed effects	Yes	Yes	Yes	Yes	Yes
Date fixed effects	Yes	Yes	Yes	Yes	Yes

### Heterogeneous treatment effects

We next explore how the treatment effect and the response to the number of ads varies with county-level characteristics. We begin by considering the percent of voters in the county who voted for Trump. (We use vote share for Trump from the 2016 election, as county-level measures were more readily available for this year. Vote shares for the 2020 election are highly correlated with those of 2016.) It is a priori unclear which types of counties should exhibit a stronger response to the treatment. One possibility is that counties with more Trump support would be more affected by the ads. This is based on the assumption that in places where more people support Trump, an ad featuring Donald Trump will be more likely to influence people.

An alternative possibility conceptualizes Trump support as part of a broader context in which people are more likely to be generally conservative. This might influence responses to our ad, because highly conservative areas are generally most resistant to vaccines and could impose the greatest anti-vaccine social pressure on the people who viewed our messages. Consistent with this possibility, in the fall of 2021, when Trump endorsed the COVID-19 vaccine or booster shots at events, in front of his strongest supporters, the conservative crowds reacted with loud boos (*22*). In addition, Bechler and Tormala (*23*) find that messages about COVID-19 vaccines are most effective when targeted toward groups with more favorable attitudes toward vaccines.

We also consider heterogeneous treatment effects by education (percent college educated) and race (percent white). Here, we come to the analysis without strong expectations, although it bears note that individuals with less educational attainment might be less likely to know that Trump supported vaccination, and thus more responsive to our message. Because our expectations have no clear direction, we report statistical significance based on two-tailed tests.

Throughout these analyses, we stress that all counties in our study had vaccination rates below 50%. Vaccination rates are strongly correlated with the moderating variables we consider in this section. So, by proxy, we are examining unrepresentative distributions of these variables. The median vote share for Trump across counties in our sample is 69.4%, the median percent of residents who are college educated is 28.7%, and the median percent white is 91.3% (see "Materials and Methods"). These medians are each more extreme than the corresponding medians computed using all U.S. counties (this is also true comparing means rather than medians).

The estimates from this analysis are shown in Table 4, where we report the difference between the effect in low relative to high counties for a given county characteristic: Trump vote share in columns 1 and 2, the percent who are college educated in columns 3 and 4, and the percent white in columns 5 and 6. We find that the ITT effect in counties with below-median Trump vote share is 258.7 vaccines higher than in stronger Trump counties, and this difference is significant at the 0.10 level in a two-tailed test under both county-level clustering and randomization inference. These results suggest that our message is highly effective in garnering a behavioral response among certain counties (those with less than 69.4% of voters favoring Trump) and not among those with more extreme proportions of Trump supporters. This result is consistent with the possibility, discussed above, that a more conservative social context would dampen message effects. In columns 3 to 6, we do not detect a statistically significant difference between the effect in less- versus more-educated counties or in whiter versus less-white counties. Together, these results suggest that, within our sample of low-vaccine counties, the most responsive counties to the message are those with strong but less-extreme Trump support.

**Table 4.** Vaccine increase per county: Heterogeneous effects and causal responses. Table reports the difference in the estimated effect in low versus high counties based on a given county characteristic. This characteristic is the 2016 Trump vote share in columns 1 and 2, the fraction of county residents with a college degree in columns 3 and 4, and the fraction of county residents who are white in columns 5 and 6. High refers to counties that are above the median level for that characteristic, and low refers to below the median, where the median is computed across counties in our sample. In odd columns, the effect is the ITT effect and in even columns it is the ACR. “***,” “**,” and “*” indicate significance (from a two-tailed test) at the 0.01, 0.05, and 0.10 levels. Standard errors, reported in parentheses below each estimate, are clustered at the county level. Randomization inference *P* values are from a two-tailed test based on 1000 permutations using the effect in low-relative-to-high counties as the randomization test statistic. Table S5 contains estimates of other coefficients from these regressions.

	% Trump		% College		% White	
	(1)	(2)	(3)	(4)	(5)	(6)
	**ITT**	**ACR**	**ITT**	**ACR**	**ITT**	**ACR**
Effect in low relative to high county	258.7* (154.5)	17.28* (11.24)	−56.76 (158.5)	4.496 (10.84)	216.3* (152.3)	12.15 (12.18)
County fixed effects	Yes	Yes	Yes	Yes	Yes	Yes
Date fixed effects	Yes	Yes	Yes	Yes	Yes	Yes
Randomization inference *P* value	0.096	–	1.00	–	0.149	–

We also repeat our heterogeneous treatment effect analysis using instead the vaccine rate within each county as the dependent variable. We again find differential effects in terms of Trump vote share: In counties that are below the median, the ITT effect is 1.775 percentage points larger than in counties with above-median Trump share. The ACR analysis suggests that an increase of 1 more ad per 100 county residents increases the percent vaccinated by 0.09 percentage points more in low-Trump-share counties than in high-Trump-share counties. Each of these effects is significant at the 0.05 level in a two-tailed test. The results are reported in table S7. Effects separated by race or education again show no statistically significant differences, suggesting that the strongest heterogeneous effects of those we examine are the Trump share results.

### Event study

Our analysis thus far compares treatment and control counties before and after the campaign began, pooling together all pre-campaign dates and similarly pooling together all post-campaign dates. This pooling does not allow us to see when precisely the vaccine uptake occurred. To examine this, we adopt an event study design that expands on our main regression analysis to estimate ITT effects at each specific date before and after the campaign.

**Fig. 3.**
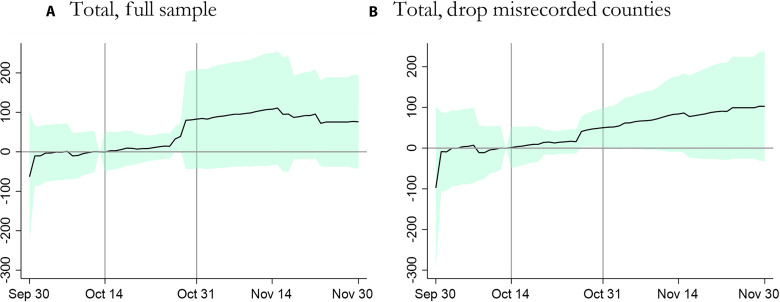
Event study with standard errors clustered at county and date levels. (**A** and **B**) Coefficients from event study regression (Eq. 4), the effect on the cumulative vaccine count up through a given date. (A) uses the full sample, and (B) drops counties that ever record a decrease in cumulative vaccine count over time. Shaded region represents 95% confidence intervals computed under two-way clustering at the county level and date level. These confidence intervals are traditional two-sided 95% confidence intervals, as a one-sided confidence interval for our test would have a tighter lower bound and no upper bound (an upper bound of infinity).

The results are shown in Fig. 3. Estimating a separate effect for each date, as we do here, can substantially reduce power. We therefore construct 95% confidence intervals (shown in the shaded region) by clustering at the date level, in addition to the county level, allowing for contemporaneous correlations across counties. As with our other results in the paper, replacing county-level clustering with state-level clustering would reduce the size of these confidence intervals. In the Supplementary Materials, we report a variant of the above event study that estimates the difference between treatment and control counties in terms of their daily vaccination count on a given date rather than the cumulative vaccination count. We also report results with state-and-date-level clustering in fig. S2.

The results suggest that the difference between treatment and control counties is not significantly different from zero before the campaign, which offers additional reassurance that our randomization worked as intended. This is a test of the parallel trends assumption required for identification in a difference-in-difference model, evidence that the number of vaccines in treatment counties—and the trend in that number—is not statistically significantly different from that of treatment counties before the start of the campaign. Figure 3 also shows that, after the campaign begins, the effect remains small initially. The cumulative effect increases near the end of the campaign and continues to increase slightly through the first 2 weeks following, peaking at around 100 vaccines—consistent with our main estimates from Table 1—at which point the cumulative effect decreases. Near the end of the sample, the difference between treatment and control counties is swamped by other noise, with the confidence intervals being quite wide and containing zero.

There are several possible explanations for the pattern of point estimates (the solid black lines) we observe in Fig. 3. First, research on the effects of advertising on behavior suggests that there are important cumulative effects of exposure in that, initially, several impressions may be needed to generate a response, after which the marginal impact of exposure declines (*24*, *25*). Second, we discovered that the CDC data itself is recorded with a lag for some counties, implying that, if the treatment did have an effect on a given date, it may appear in CDC data on a later date. For example, in some observations in the CDC data, a county’s cumulative vaccine count jumps up by over 30,000 in a single day—for one such county, this jump corresponds to about one-third of the entire county population—an implausibly large amount for a single day, but consistent with some counties updating their vaccine count infrequently and, therefore, in batches. In any event, the fact that our point estimates are similar regardless of whether these counties are included suggests that the data errors are uncorrelated with the treatment assignment and only introduce statistical noise, not bias, into our estimates. We perform additional robustness checks on this subsample in SM Section A.

Third, it is possible that those affected by the ad chose to schedule vaccine appointments for several days or a week in the future rather than getting vaccinated on the day they viewed the ad. Even for vaccines administered as walk-ins, rather than scheduled appointments, it is plausible that the advertising campaign had an effect with a lag, with viewers’ changing attitudes being reflected in actual vaccinations only after several days or a week of the attitude change. For example, viewers may decide, consciously or not, “I won’t go out of my way to get the vaccine today, but I’ve decided to go ahead with getting vaccinated next time I find myself in a drugstore.”

Finally, it may be that nationwide changes in vaccine policy, which affected both treatment and control counties, had differential impacts on treatment counties due to the priming of the advertising campaign. The key change in vaccine policy that occurred during our sample period was that children ages 5 to 11 became eligible for the vaccine on November 2. Information about this policy change was leaked slowly over the week preceding the change, with an official announcement from the CDC released on November 2 announcing the November 4 eligibility date. One possibility is therefore that the changing guidelines around childhood vaccination, and attention to these changes, can account for some of the reason that the treatment effect was larger near the end of the campaign.

## DISCUSSION

The COVID-19 vaccines proved effective, substantially reducing the chances of hospitalization and death among those who took them. Remarkably, politics became a major obstacle to their adoption and use. Large segments of the population declined to be vaccinated, with partisanship exerting the largest effect. Hesitancy in the U.S. proved especially high, with rates of vaccination barely among the top 50 countries in February 2022 (*26*). Beyond leaving a disproportionate number of Republicans more vulnerable to the virus, greater hesitancy comes with negative externalities: the prevalence of breakthrough infections as well as the risk to immunocompromised individuals vulnerable to COVID infections even if vaccinated. As many have observed, a tragedy of the COVID-19 pandemic is the extent to which protective measures became tangled in Americans’ political identities, which led to deaths and suffering that could have been avoided.

But if politics characterizes one aspect of the problem, it might also be part of a solution. We find that positioning Donald Trump and Fox News as counter-stereotypical messenger is likely a cost-effective way to overcome hesitancy among people who still had not been vaccinated, months after the vaccines became widely available. An important caveat to our results is that we do not have conclusive evidence that it was indeed the counter-stereotypical nature of the message that led to its effectiveness; we may have simply designed an effective ad, and it is possible that other (nonpolitical) ads would have a similar positive impact. Unfortunately, there is scant experimental evidence of the effectiveness of advertising on COVID-19 vaccine uptake to which our effects can be compared. Regardless of the specific mechanism, our point estimates suggest higher number of vaccines per dollar spent than other interventions that have been studied.

Are our findings scalable? We believe they are. With seasonal flu vaccines—and immunization attitudes generally—starting to show signs of increased partisan schism in the wake of the COVID-19 pandemic (*27*), support from party leaders for vaccinations may represent a potent tool that public health messengers can use. Insofar as vaccinations continue to be politicized, this research provides a model for political messaging as an important public health tool in future pandemics.

Whether the dividing line is politics or something else, our study suggests that public health proponents might do well to reflect on messengers whose voices might carry special weight among target populations. For example, other research finds that a pro-masking message from a military general increases support for masking among political conservatives (*28*). The results we report here help corroborate this theme. We think it represents a promising route to overcome resistance and, in turn, save lives.

## MATERIALS AND METHODS

### Message considerations

Previous research has demonstrated that message persuasiveness rarely hinges on argument quality, because evaluating quality requires effortful information processing that many people avoid (*29*, *30*). Instead, people tend to rely on mental shortcuts, such as beliefs about a source’s credibility, to decide whether to accept or reject new information (*31*). Donald Trump stands out as distinctive in this respect. Original survey data we collected in the months following the pandemic’s onset revealed that members of our target audience (unvaccinated Republicans) persistently had greater confidence in vaccine advice coming from Donald Trump than in advice coming from more traditional sources, such as their personal doctor or the scientific community (Table 5).

**Table 5.** Percent of unvaccinated Republicans expressing a “great deal of confidence” “when it comes to advising you on taking the covid-19 vaccine.” Cell entries represent the percentage of respondents in a national survey indicating they have a great deal of confidence in vaccine advice coming from each of the indicated sources. This table relies on two different waves of the survey, March/April (sample size 422) and August/September (sample size 387). See SM Section F for details on question wording and the sampling approach.

	March/April	August/September
Donald Trump	39	40
Joe Biden	10	8
Anthony Fauci	14	11
Your personal doctor	35	23
Scientific community	16	11

Because Republicans have become identified with skepticism about the severity of the COVID-19 virus and COVID vaccines, a pro-vaccine message from the leader of the Republican Party qualifies as counter-stereotypical—and counter-stereotypical messages have been shown to evoke more effortful mental processing (*10*). Recent survey experiments conducted on a convenience sample found that a vaccine message from Donald Trump successfully increased vaccine intentions among Republicans (*7*, *8*). We build on those insights, but rather than use self-reported attitude surveys as an outcome measure, we focus on actual vaccination behavior.

A pro-vaccine endorsement from Donald Trump that is associated with Fox News should further enhance the counter-stereotypical nature of the message. Fox News personalities have been skeptical of Fauci and many COVID vaccine efforts. Moreover, previous research has demonstrated the persuasive effects of Fox News on Republican attitudes broadly, as well as on viewers’ attitudes toward pandemic guidelines in particular (*32*, *33*).

### The PSA

The PSA (which can be viewed at https://www.youtube.com/watch?v=INH-CmCgIYs) includes four separate video clips—the first and third from a Fox 13 News Utah (a local station) segment recorded on 16 March 2021, the second from a phone interview between Donald Trump and anchor Maria Bartiromo recorded on the Fox News Channel (the nationwide cable TV channel) from the same date, and the fourth from a social media post of Ivanka Trump from the spring of 2021. We hired a professional video editor to combine these clips and overlay them with an engaging soundtrack.

Knowing that many users might opt to stop the PSA from playing as soon as possible, it was imperative that news of Trump’s endorsement occur immediately. Within the first 3 s of our ad, the Fox 13 Utah anchor says, “Donald Trump is urging all Americans to get the COVID-19 vaccine.” The rest of the PSA unfolds as follows:

1) Seconds 4 to 12: Donald Trump speaking on phone interview on Fox News with Bartiromo, while Bartiromo nods in agreement: “I would—I would recommend it, and I would recommend it to a lot of people that don’t want to get it, and a lot of those people voted for me, frankly.”

2) Seconds 13 to 19: Fox 13 News Utah anchor, with footage of the Trumps at White House: “Both Trump and former First Lady Melania Trump did receive their vaccines privately in January at the White House.”

3) Seconds 20 to 23: Screen text quote from Ivanka Trump, with still shot of her getting the vaccine: “Today I got the shot. I hope you do too.”

4) Seconds 24 to 27: Black screen with white print: “Your vaccine is waiting for you.”

Our choice of wording for this final frame was driven by evidence that, for flu vaccines, patients are most responsive to framing suggesting that a vaccine is reserved for them (*34*).

The specific YouTube setting we adopted required users to watch the first 5 s of the PSA before being allowed to skip. Hence, viewers, even if only involuntarily, heard that Trump was urging all Americans to get the vaccine. A screenshot taken at 2 s into the ad appears in Fig. 1. One second later, viewers saw Fox News’ familiar news anchor, Bartiromo, and the official Fox News stamp. For viewers who had the sound on their devices muted, on screen text delivered the message. The Fox Utah news story prominently displays, “Trump encourages getting COVID-19 vaccine” on the screen, and the Fox News story shows, “Trump on the success of operation warp speed.” We included closed captions of all spoken words to increase the likelihood that viewers would absorb the message. We also embedded a link in the bottom left corner of the ad, allowing viewers to click to see the full Fox News interview with Bartiromo. In the bottom right, a box displays a countdown of the number of seconds until the user can skip the ad (showing “3″ in Fig. 1).

### How YouTube’s advertising platform works

For each advertising slot—someone watching a YouTube video—Google runs an instantaneous auction, bidding on behalf of each advertiser, to determine which advertiser’s content will be shown (like search or display ads). YouTube’s advertising platform (Google Ads) allowed us to select a target population (our treatment counties) and to specify our willingness to pay for 1000 ad impressions (CPM), known as a “mille” in advertising lingo. We use CPM to gauge the cost effectiveness of our campaign. An advertiser is more likely to win an auction if she is willing to pay more than other advertisers and if Google predicts that, for a given user, the advertiser’s content is more likely to generate user engagement (which Google defines as watching at least 10 s of the ad). As we describe below, these features generated widely varying exposure to our ad across different counties, as Google’s algorithm dynamically adjusted as it learned which types of users were more likely to engage with the ad.

### Selection of treatment and control counties

We designed our experiment to administer ads such that they would be concentrated in areas with low vaccination rates while also facilitating our ability to estimate effects on actual vaccine uptake at the county level, the smallest geographic unit for which vaccine administrative records are widely available. Specifically, we excluded counties with vaccination rates above 50% (full vaccine series complete, according to CDC records as of 28 September 2021). We also excluded counties with populations above 1 million (45 counties). Aside from being culturally distinctive (e.g., large cities) and expensive to target, these had the potential to exert disproportionate influence on our results because the distribution of county populations has a long right tail. We also excluded the following other areas that we deemed inappropriate for our study. First, we excluded several—mostly uninhabited—Alaska Census areas. These are not conducive to YouTube targeting, as they are not counties. We excluded all counties in Texas (254), as these counties are not consistently included in CDC records (our source for measuring vaccine uptake). Third, we exclude Washington, D.C., given its unique cultural status.

These exclusions left us with 2168 counties eligible for the study. We divided these into quintiles according to (i) population and (ii) percentage of the population vaccinated and then created strata defined by the intersections of these two classifications. The creation of these strata happened before we chose to exclude high-vaccine counties from our experiment. Applying this restriction dropped the top quintile in terms of vaccination rates, leaving us with 20 strata created by the intersection of the two classifications. We then randomly assigned counties to treatment or control, blocking by the 20 strata, using the software developed by Blair *et al*. (*35*). This procedure resulted in 1083 counties assigned to receive ads, and 1085 retained as a control group.

Table 6 provides summary statistics for the counties included in our study—separately for counties assigned to treatment and control conditions. Treatment and control groups are closely balanced on prior percentage of the population vaccinated (diff = 0.01, SE = 0.39, *P* = 0.98) as well as population (diff = −3216, SE = 3757, *P* = 0.39). Table 6 also shows that treatment and control counties are well balanced on the share of the vote received by Trump, the percent of the population who are college educated, the percent who are white, and the level of internet access, although we did not intentionally target balance along these variables.

**Table 6.** Descriptive statistics by county. Descriptive statistics at the county level. Top panel shows statistics for all counties, middle panel for treatment counties, and bottom panel for control counties. Vaccine first doses before campaign correspond to the vaccine count in the county on October 13. A county’s engagement rate is the number of 10-s (or longer) views of the ad, divided by the number of ad impressions the county received, multiplied by 100. View rate is the number of complete views of the ad divided by the number of impressions, multiplied by 100. Click rate is the number of times the Fox News story link was clicked, divided by the number of ads, multiplied by 100. CPM is the average cost (in dollars) of purchasing 1000 ad impressions in the county. Data on vote shares come from the MIT Election Data Science Lab (https://electionlab.mit.edu/data). Data on internet access come from the FCC (https://www.fcc.gov/form-477-county-data-internet-access-services). Internet access is defined as having a fixed high-speed connection over 200 kbps in at least one direction. Data on county population and county-level education and racial demographics come from USAfacts.org and the U.S. Census website.

	Mean	Standard deviation	Min	Median	Max
**All counties**					
County population (10,000s)	4.72	8.75	0.0463	2.16	99.9
Vaccine first doses before campaign	24,123	48,655	92	10,027	591,758
Trump vote share	67	13	10.5	69.4	96
Percent college educated	28.7	7.92	5.95	27.9	61.6
Percent white	83.7	17.1	8.33	91.3	100
Fraction households with internet	0.7	0.142	0.22	0.7	2.12
Number of households with internet (10,000s)	1.39	2.73	0	0.6	31.6
Number of counties	2,168				
					
**Treatment counties**					
County population (10,000s)	4.56	8.12	0.0463	2.08	99.9
Vaccine first doses before campaign	23,282	45,442	92	9,704	591,758
Trump vote share	67	13	10.5	69.4	96
Percent college educated	28.8	8.04	5.95	28	61.6
Percent white	83.7	17.1	8.33	91.5	99.8
Fraction households with internet	0.7	0.145	0.22	0.7	2.12
Number of households with internet (10,000s)	1.34	2.45	0	0.6	27.9
Number of ads	10,679	25,245	0	3,764	346,089
Number of ads per 100 residents	19.3	9.17	0	19	160
Engagement rate	41.3	3.32	0	41.1	63.3
View rate	12.4	1.49	0	12.3	25
Click rate	0.0838	0.0769	0	0.0776	1.14
Cost (dollars) per 1000 ads (CPM)	8.53	0.406	0	8.55	10
Number of counties	1,083				
					
**Control counties**					
County population (10,000s)	4.88	9.33	0.0465	2.24	96.5
Vaccine first doses before campaign	24,954	51,643	216	10,435	530,314
Trump vote share	67.1	13	13.6	69.4	91.8
Percent college educated	28.5	7.81	8.68	27.7	60.3
Percent white	83.7	17	11.3	91.1	100
Fraction households with internet	0.699	0.14	0.3	0.7	1.86
Number households with internet (10,000s)	1.45	2.97	0	0.6	31.6
Number of counties	1,085				

Among our 1083 treatment and 1085 control counties, 136 (69 treatment and 67 control) ended up reporting no vaccine count data to the CDC during our sample period. Our regression analyses therefore use the 1014 treatment counties and 1018 control counties with nonmissing CDC data. This set of counties spans 43 states.

### Ad campaign characteristics

Online advertising often results in low signal-to-noise ratios, requiring very large ad campaigns to detect an effect even in carefully designed randomized control trials (*11*, *36*). Fortunately, our ad budget was substantial. The total budget we spent on ads was $99,009.51. We spent the remainder of our original $100,000 budget in early testing to learn the platform’s features. Of the total $99,009.51 spent during the actual experiment, $96,408.56 was spent in counties that reported vaccine data to the CDC.

As described above, for any ad shown to a user on YouTube, an auction determines which advertiser’s ad gets displayed. Google bids on behalf of each bidding advertiser, who submits target bids—a price that a bidder would pay, on average, for 1000 impressions. We set our initial target CPM to $7.43, the level at which YouTube advertising algorithms and staff recommended. Between October 14th and 17th, we spent approximately $7500 per day on ads. As the campaign progressed, and the pool of users to which Google’s algorithm sent our ads changed dynamically, this initial target price proved too low to place our ad and spending dropped to between $2000 and $3000 on the 18th and 19th. Increasing our CPM target price to $10 allowed spending to surge above $8000 per day from the 20th to the 24th. For the complete duration of the campaign, our realized average CPM was $8.55.

The campaign ran from October 14 through October 31, with ads steadily rolling out over this period, as shown in Fig. 2A. We initially avoided any targeting of specific demographic groups other than excluding minors. After the first 10 days of the campaign, however, we observed in Google Ads tools that users ages 18 to 24 were receiving a disproportionate share of ads. In response, we excluded this age category for the last week of the campaign. Combined, we purchased a total of 11,573,574 impressions. These ads were delivered to 6,079,732 distinct viewers, with the average user seeing the ad 1.9 times and no one seeing it more than four times.

Table 6 shows that the average treatment county received 10,679 ad impressions—19 for every 100 residents. Table 6 also shows statistics of the county-level engagement rate, which in Google Ads tools parlance refers to the percent of ad instances in which users watched at least 10 s of the ad (41% in the average county). The view rate is the percent of cases where viewers watched the full 27 s of the ad (12% of viewers in the average county). The click rate refers to the percent of viewers who clicked on the link below the ad that took the user to the original Fox News story (fewer than 1% of viewers in the average county).

Google Ads tools also allow us to observe characteristics of YouTube viewers. We summarize these characteristics in table S8. The ad was shown to nearly twice as many males as females, and to more than twice as many nonparents as parents. The rate of viewing the full ad was roughly constant at about 11 to 13%, regardless of a user’s characteristics (gender, age, income, or parental status). Engagement rates hovered around 40% for most users, with users ages 18 to 24 being slightly less likely to watch at least 10 s. Users whom YouTube knows less about (marked with “Unknown” for a given characteristic in table S8) are also less likely to engage.

We also obtain from Google Ads detailed information on the outlets through which users viewed the ad, which we summarize in table S9. Panel D shows that 52% of ads appeared on phones, 30% on television screens (e.g., via Roku or Apple TV), 13% on tablets, and 4% on computers. We find the viewers watching on TV screens tended to watch much more of the ad—46% watched at least 10 s, compared to only 37% among mobile phone users. Table S9A shows that the PSA was placed on 150,284 distinct YouTube channels, several hundred websites, and 10,072 mobile apps (largely games). Figure 2B plots YouTube channels ranked by the number of times our ad displayed on each channel. Of these YouTube channels, the main Fox News channel hosted our ad the most—over 200,000 times, which is 3 times the quantity on Forbes and 10 times that on NBC News YouTube channels. Several other channels with the word “fox” in their title (such as Fox News Business) also hosted the ad—270,000 ads in all appeared on such channels. The ad also appeared on other channels with conservative leanings, such as Glenn Beck and The Blaze, as well as outlets not supportive of Trump, such as Saturday Night Live (each of these channels had over 3000 ad impressions), and many local news stations (Fig. 2B).

Google’s tools also show the specific YouTube postings to which our ad was attached for a given channel. For the Fox News channel, these include segments by cable news personalities such as Laura Ingraham, Greg Gutfeld, Tucker Carlson, Judge Jeanine Pirro, Sean Hannity, Jesse Waters, and The Five. For example, fig. S3 shows a screenshot of a Jesse Waters YouTube segment titled “Biden’s lost touch with reality.” Our ad was attached to this video 2740 times.

We emphasize here that the outlet (YouTube channel) and specific video segments were not choice variables in our design; Google’s algorithm chose to place our ad on these YouTube channels and videos based on its predictions of user engagement, a highly convenient feature for our goal of reaching a vaccine-hesitant audience using a counter-stereotypical messenger.

### Variation in ad exposure across counties

Google Ads metrics records the number of ad impressions displayed in each county over the duration of the campaign. Because of the ad auction features described above, our campaign’s budget was spent asymmetrically across our treated counties. Figure 2C shows a histogram of the number of ads received by each county, omitting the top 5% for readability. Some counties received more than 40,000 ads (with a maximum of 346,089), while some received far fewer (including five small, treatment-assigned counties that received zero). This is because YouTube does not treat counties as separate blocks, each to be assigned some ad exposure. Instead, the whole list of counties is taken to be a single target audience and YouTube attempts to serve the ad to the users within that audience who are most likely to engage with the ad. The average county saw 10,679 ads, with a standard deviation of 25,245 (Table 6). Table S2 demonstrates that, among treated counties, the number of ads a county receives is significantly positively correlated with county population and the level of internet access in the county.

**Fig. 4.**
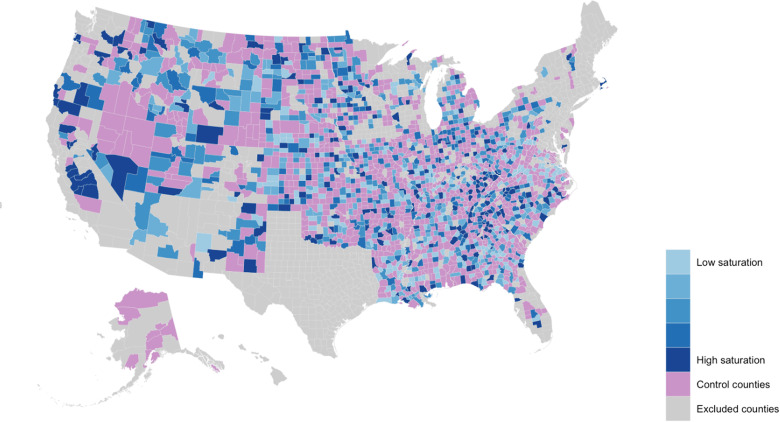
Geographic distribution of advertising campaign. Gray regions are counties that were excluded (highly vaccinated, large population, or poor CDC records). Purple shows control counties. Blue shows the distribution of ads at the county level for treatment counties, with the five shades of blue corresponding to five quintiles of ad displays per resident.

Figure 2D normalizes the number of ads by the county population, showing a histogram of the number of ads per 100 residents in the county separately for large (those with a population above the median) versus small counties, omitting the top 5%. These numbers range from 0 to nearly 30 ads per 100 residents for both sets of counties (with a maximum of 160; see Table 6), demonstrating that, even accounting for population differences, the number of ads is quite variable across counties, with the number of ads per capita being higher in larger counties. Figure 4 shows that this variation has no obvious geographical bias: High- and low-saturation counties are distributed more or less evenly throughout the entire United States.

### Empirical approach

We pre-registered our analysis plan via the Open Science Framework. (Our pre-registration plan can be viewed at https://osf.io/m9yhn/?view_only=c0d43e87224649e88b671eafddb22df8.) We discuss reasons for specific departures from our pre-registration in SM Section E.

Our pre-registered dependent variable is the cumulative number of COVID-19 vaccine first doses administered in each county up through a particular date. We focus only on vaccine recipients who are 12 or older, as children ages 5 to 11 only became eligible after our campaign ended (in early November 2021). An observation in our analysis is a given county on a given date.

Let *y_it_* denote the cumulative number of COVID-19 vaccine first doses among residents ages 12 and older in county *i* up through date *t*, obtained from https://data.cdc.gov/Vaccinations/COVID-19-Vaccinations-in-the-United-States-County/8xkx-amqh. Our analysis encompasses dates from 1 month before the campaign to 1 month after, which includes 151,945 county-date observations. (See SM Section C for an analysis of alternative time windows.) Let Treat*_i_* be an indicator variable equal to 1 if county *i* is a treated county and 0 otherwise. Let Post*_t_* be a binary variable equal to 1 if date *t* occurs on or after October 14, the start date of the campaign. Let Population*_i_* be the population of county *i* (in units of 10,000). We estimate the following difference-in-difference regression:

$$y_{it}=\alpha +\lambda_{t}+\gamma_{i}+\beta ({\mathrm{Treat}}_{i}\times {\mathrm{Post}}_{t})+\eta ({\mathrm{Population}}_{i}\times {\mathrm{Post}}_{t})+\varepsilon_{it}$$ (1)

The variable λ*_t_* is an effect for date *t*, capturing nationwide trends in vaccinations on a given date, and γ*_i_* is a county effect, capturing time-invariant differences in vaccination counts across counties. λ*_t_* and γ*_i_* also absorb the main effects Treat*_i_,* Population*_i_*, and Post*_t_*. The interaction term Population*_i_* × Post*_t_* allows for the possibility that the cumulative vaccine count grows at a different rate over time in counties of different sizes. The inclusion of this interaction term represents a departure from our pre-registered regression analysis plan. We discovered the importance of controlling for differential growth rates by county population only after the campaign was complete. SM Section C discusses this in more detail and, for transparency, reports our pre-registered specifications.

Our estimation algorithm to incorporate these two-way fixed effects relies on Baum *et al*. (*37*). The residual ɛ*_it_* includes all unobserved factors affecting the number of vaccines administered in a particular county on a given date. Not all counties in the treatment group received ads, and for those that did receive ads, the exposure varied widely across counties. Thus, the primary coefficient of interest, β, is the ITT effect.

One important assumption underlying our analysis is the stable unit treatment value assumption (SUTVA), which requires that a county’s treatment status does not affect the potential outcomes of other counties. This assumption could be violated, for example, if some people view the ad in a treatment county and then cross county borders into a control county to get the vaccine. If present, this type of violation would lead to our estimates of the ITT effect being understated.

In some regressions, we modify Eq. 1 to include flexible interactions of county population with dates in our sample period. These are implemented by replacing the product of η and Post*_t_* in η(Population*_i_* × Post*_t_*) in Eq. 1 with

$$\sum_{\tau \in T}\eta_{\tau }1_{t=\tau }+\eta_{\underline{t}}1_{t<\underline{t}}+\eta_{\bar{t}}1_{t>\bar{t}}$$

where $T=\{\underline{t}+1,\underline{t}+2,\ldots ,t^{*}-1,t^{*}+1,\ldots ,\bar{t}-2,\bar{t}-1\}$ is a window of dates defined as 13 days before the campaign up through 13 days after, with t^*^, the date immediately preceding the campaign, being omitted. Thus, $\underline{t}$ is September 30, $\bar{t}$ is November 14, and t^*^ is October 13. **1***_E_* is an indicator equal to 1 if the event *E* is true.

### Inference considerations

We apply one-tailed hypothesis tests in most specifications for two reasons. First, low signal-to-noise ratios make measuring the effects of advertising notoriously difficult. One recent contribution concluded that “informative advertising experiments can easily require more than 10 million person-weeks, making experiments costly and potentially infeasible” (*11*). Another recent meta-analysis of 40 field experiments studying persuasion effects of political campaign advertisements resulted in an average effect estimate of zero (*12*). These challenges are exacerbated by the fact that, in our setting, we only have county-level outcome data, likely rendering our study underpowered from the perspective of a two-tailed test. Second, and more importantly, our expectations are clearly directional, evidenced by the obvious fact that it would be unethical to conduct a study that we believed might discourage people from receiving life-saving vaccines. With directional expectations, a one-tailed test is the correct approach, since a two-tailed test would lead to an inflated type 1 error rate. Moreover, a negative treatment effect in Table 1, for example, would arise only if there exist viewers who would have been vaccinated absent seeing our ad but who decide to not get the vaccine because of the ad. We believe this is implausible, making a one-tailed test the natural choice. For analyses where our expectations are not directional (our analysis of heterogeneity in treatment effects), we instead apply two-tailed tests.

We adopt two main approaches to conducting inference throughout: randomization inference and county-level clustering. In randomization inference (*38*), the researcher uses Monte Carlo methods to simulate the distribution of effect sizes that arise under the “sharp null” hypothesis (i.e., a treatment effect of zero for all units). To implement this, we follow our stratified random sampling routine to create a randomly assigned placebo treatment status for each county and reestimate our effects of interest with this placebo treatment status. We repeat this exercise 1000 times, reassigning placebo treatment status each time. We then compute *P* values as the fraction of cases (out of the 1000) where the estimate effect is larger than the effect estimated with the correct treatment assignment. We apply this approach to all ITT effects in the paper.

It bears emphasis that the assumptions underlying randomization inference differ from those of asymptotic approaches (our clustering approaches): Where the asymptotic approaches test the null hypothesis that the average treatment effect across units is zero, randomization inference tests the more restrictive hypothesis that the effect is zero for every unit, which recent work of Wu and Ding (*39*) shows can yield *P* values that are larger or smaller than those corresponding to a hypothesis test of the average treatment effect. The virtue of the approach is that it yields exact finite-sample inference for the sharp null hypothesis without requiring us to take a stance on a level of geographic clustering.

For our second approach—clustered standard errors—our pre-registration plan stated an intention to estimate uncertainty with state-level clustering (43 clusters) to allow for the possibility that county-level residuals may be correlated within a state. The choice of the level at which to cluster standard errors is the subject of ongoing research in econometrics. It is known that bias can result from having too few clusters on one hand or from not clustering at an aggregate enough level on the other. However, the question of what constitutes “too few” is unresolved; some researchers point to 50 as a reasonable threshold, others to 20 (*40*). According to Cameron and Miller (*40*), “the consensus is to be conservative and avoid bias and use bigger and more aggregate clusters when possible, up to and including the point at which there is concern about having too few clusters. For example, suppose your dataset included individuals within counties within states, and you were considering whether to cluster at the county level or the state level. We have been inclined to recommend clustering at the state level. If there was within-state cross-county correlation of the regressors and errors, then ignoring this correlation (for example, by clustering at the county level) would lead to incorrect inference.“

Our pre-registered preference for state-level clustering was driven by this conventional wisdom. More aggregate clustering typically results in larger standard errors, but, as highlighted by Cameron and Miller (*40*), it is possible for more aggregate clustering to reduce standard errors when residuals are negatively correlated across observations in a more aggregate cluster. Abadie *et al*. (*41*) critique the conventional view, presenting arguments for clustering at the level at which randomization occurs (the county, in our case).

We find this to be the case in our setting, where the standard errors on our effects of interest are smaller under state-level clustering or stratum-level clustering than under county-level clustering. Table S10 demonstrates these results. There, we find that our results from Table 1 are significant at the 0.01 level when we apply state-level clustering and the ACRs from Table 1 are significant at the 0.05 level when we apply stratum-level clustering. Two-way clustering, following Cameron and Miller (*40*), combining geographic clustering with date-level clustering to allow for possible correlations across counties on a given date, makes little or no difference to our estimated standard errors. In the end, we adopted the most conservative standard errors from this analysis—county-level clustering—to avoid overstating the strength of our findings.

### Assessing causal response to treatment intensity

We also move beyond the ITT effect to analyze how the number of ads a county receives affects the number of vaccines in the county, referred to as the ACR (*42*). To measure this effect, we implement the IV design proposed by Angrist and Imbens (*42*), instrumenting for the number of ads in each county using the county’s random assignment to treatment or control.

The reason for this design is important: Unlike assignment to treatment or control status, the number of ads a county receives is not randomly assigned, but rather arises from the black box of Google’s machine learning predictions of viewers’ likelihood of engaging with the ad together with variation in competition for ad auctions. As such, it is possible that Google sends more ads to counties where viewers are more likely to be receptive, and hence a standard ordinary least squares (OLS) regression treating the number of ads as randomly assigned would not yield an unbiased estimate of the effect of ad exposure. (This does not bias the estimate of ITT effect, only the naïve estimate of the causal effect of an increase in ad exposure.) The IV approach, on the other hand, exploits the random assignment to restore a causal estimate of the response to an increase in the number of ads. Our setting also falls into the special case of one-sided noncompliance, meaning here that some treatment counties received no ads, but no control counties received ads (*43*). In this case, our effect corresponds to the ACR for treated counties.

In our difference-in-difference regression framework, our IV regression is as follows

$$y_{it}=\alpha +\lambda_{t}+\gamma_{i}+\delta ({\mathrm{Ads}}_{i}\times {\mathrm{Post}}_{t})+\eta ({\mathrm{Population}}_{i}\times {\mathrm{Post}}_{t})+\varepsilon_{it}$$ (2)

where we instrument for Ads*_i_* × Post*_t_* using Treat*_i_* × Post*_t_*, with Ads*_i_* denoting the number of ads received by the county, measured in units of 1000 impressions. Thus, the estimate of δ represents the ACR for treated counties from an additional 1000 ad impressions. We rely here on the estimation algorithm of Correiga (*44*).

### Assessing heterogeneous effects

As discussed in Results, we sought to examine the extent to which treatment effects were moderated by county-level support for Donald Trump, education (percent with a college education), and race (percent white). For each of these three characteristics, we compute the median across counties, and we let *W_i_* be a dummy variable equal to 1 if county *i* is below the median value for that characteristic. We then estimate regressions of the following form

$$y_{it}=\alpha +\lambda_{t}+\gamma_{i}+\beta ({\mathrm{Treat}}_{i}\times {\mathrm{Post}}_{t})+\phi ({\mathrm{Treat}}_{i}\times {\mathrm{Post}}_{t}\times W_{i})+\psi (W_{i}\times {\mathrm{Post}}_{t})+\eta ({\mathrm{Population}}_{i}\times {\mathrm{Post}}_{t})+\varepsilon_{it}$$ (3)

In Eq. 3, β represents the ITT effect for above-median counties and β + ϕ represents the effect for below-median counties, and ϕ represents the difference between the below-median and above-median effect. We again estimate the ACR using IV. Specifically, we replace Treat*_i_* in Eq. 3 with Ads*_i_* and we instrument for (Ads*_i_* × Post*_t_*) and (Ads*_i_* × Post*_t_* × W*_i_*) using (Treat*_i_* × Post*_t_* × W*_i_*) and (Treat*_i_* × Post*_t_*).

### Event study analysis

To conduct an event study analysis, we run the following regression

$$y_{it}=\alpha +\lambda_{t}+\gamma_{i}+{\mathrm{Treat}}_{i}(\beta_{\underline{t}}1_{t\leq \underline{t}}+\sum_{\tau >\underline{t},\tau \neq t^{*}}\beta_{t}1_{t=\tau })+{\mathrm{Population}}_{i}(\eta_{\underline{t}}1_{t\leq \underline{t}}+\sum_{\tau >\underline{t},\tau \neq t^{*}}\eta_{t}1_{t=\tau })+\varepsilon_{it}$$ (4)

where the date $\underline{t}$ is September 30 and t^*^ is October 13, the day before the start of the campaign. Thus, this specification estimates a date-specific effect for each date after September 30, and a single coefficient pooling together dates before that.

We also estimate a variant of the above event study that controls for *y*_*it* − 1_, the lagged vaccine count within county *i*, on the right-hand side, as follows

$$y_{it}=\alpha +\delta y_{it-1}+\lambda_{t}+\gamma_{i}+{\mathrm{Treat}}_{i}(\beta_{\underline{t}}1_{t\leq \underline{t}}+\beta_{\bar{t}}1_{t\geq \bar{t}}+\sum_{\tau >\underline{t},\tau \neq t^{*}}\beta_{t}1_{t=\tau })+{\mathrm{Population}}_{i}(\eta_{\underline{t}}1_{t\leq \underline{t}}+\eta_{\bar{t}}1_{t\geq \bar{t}}+\sum_{\tau >\underline{t},\tau \neq t^{*}}\eta_{t}1_{t=\tau })+\varepsilon_{it}$$ (5)

Equation 5 also bins together dates falling within the last 2 weeks of the sample (those on or after November 14, which is denoted $\bar{t}$). This regression estimates the difference between treatment and control counties in terms of their daily vaccination count on a given date rather than the cumulative vaccination count.

## Supplementary Materials

### This PDF file includes:

Supplementary Text Sections A through D Figs. S1 to S3 Tables S1 to S12
