# Supplementary material for: Counter-stereotypical messaging and partisan cues: Moving the needle on vaccines in a polarized United States
Source: Sci Adv. 2023 Jul 19;9(29):eadg9434. doi: 10.1126/sciadv.adg9434 (PMC10355821; doi:10.1126/sciadv.adg9434)
Supplement: Supplementary file 1 — Supplementary Text Sections A through D Figs. S1 to S3 Tables S1 to S12 [file sciadv.adg9434_sm.pdf]

Science Advances
AAAS

Supplementary Materials for

Counter-stereotypical messaging and partisan cues: Moving the needle on vaccines in a polarized United States

Bradley J. Larsen *et al.*

Corresponding author: Timothy J. Ryan, tjr@email.unc.edu

Sci. Adv. **9**, eadg9434 (2023)
DOI: 10.1126/sciadv.adg9434

This PDF file includes:

Supplementary Text Sections A through D
Figs. S1 to S3
Tables S1 to S12

Supplementary Text

A: Robustness Checks for Core Results

We consider two robustness checks for our main results. During our sample period, two types of obvious mis-recordings occur in the CDC county-level daily data. The first is that some counties show a decrease from one day to the next in their cumulative vaccination count. For example, the data can show that a given county has administered a total of 34,500 COVID-19 vaccine first doses since the beginning of the vaccine's availability, up through and including date *t*, and then show that this number decreases to 33,300 on the following day, which is impossible. The second type of error is simply that the vaccine count is missing for some dates during our sample for certain counties. We replicate our main analysis dropping any counties with misrecorded CDC data. The results are shown in Table S1. Columns 1-2 replicate the ITT analysis of Table 2 but drop counties that have reported decreases in cumulative vaccine counts, and columns 5-6 instead drop counties that have missing vaccine counts for any date in the sample period. Table S1 shows that the effects are positive, as in Table 2, and slightly larger in magnitude. The results in columns 1-2 have a similar level of statistical significance to those in Table 2, although columns 5-6 are no longer significant at the 0.10 level.

In Table S1, columns 3-4, we report the estimated ACR after dropping from our sample any counties with misrecorded CDC data. Here we observe an effect that is similar in magnitude and significance. In columns 7-8, where we drop counties with CDC data missing for at least one date, the estimated ACR is similar in magnitude but no longer significant.

We also repeat our instrumental variables regressions from Table 1 using a heterogeneous first-stage regression. For this analysis, we create five indicator variables for whether a county is above or below the median in terms of five characteristics: percent Trump voters, percent college educated, percent white, percent with internet access, and county population. As shown in Table S2, these five county-level characteristics are all significantly correlated with the number of ads a county receives, making them candidates for a heterogeneous first stage regression. We group counties into 32 (2^5) different groups based on the realizations of these five indicator variables and interact these 32 variables with $Treat_i \times Post_t$. We then repeat the instrumental variables regression of equation (2) using these 32 variables as excluded instruments for $Ads_i \times Post_t$ rather than only using $Treat_i \times Post_t$ as the excluded instrument. As additional included instruments (included in the first and second stage) we use the interactions of $Post_t$ with the indicators for below median Trump voters, percent college educated, percent white, and percent with internet access. Note that the interaction of $Post_t$

with county population is already included (linearly). The results of this IV regression with a heterogeneous first stage are shown in Table S12. We find similar estimates to those in Table 1, with an average causal response of about 10.9 vaccines to an increase of 1,000 ads, regardless of how we control for differential trends across population.

B: Day-focused Event Study Analysis

Figure S1 contains an alternative to our event study analysis, where we use as the outcome the number of vaccines administered on a given date in a given county (rather than the cumulative number of vaccines so far in the county). These daily level results are more noisily estimated than the cumulative results. However, consistent with Figure 3.A, Figure S1.A shows that differences between treatment and control counties prior to the start of the campaign were small and largely insignificant. We observe large point estimates later in the campaign (in the last week of October), and then a leveling off at about 10 vaccines per day in the final days of the campaign and the two weeks afterward.

Upon investigation, we learned that the large spike on October 29 seen in Figure S1.A is driven by counties with misrecorded CDC data, in which the county records a *decrease* in its cumulative vaccination count over time, as discussed in SM Section A. Figure S1.D omits these counties, and the October 29 spike disappears. We also report all of our event study specifications with state- (rather than county-) and-date level clustering, and display the results in Figure S2. Here we see a similar pattern to that in Figures 3 and S1 but with tighter confidence intervals.

C: Departures from Pre-Registration Plan

We pre-registered our analysis plan via the Open Science Framework at https://osf.io/m9yhn/?view_only=c0d43e87224649e88b671eafddb22df8. Our analysis described in the body of the paper follows this pre-registered plan to the extent possible. Specifically, our pre-registered dependent variable is the number of vaccines administered in each county up through a given date. Our pre-registration plan also stated that we would analyze effects of our campaign through difference-in-difference OLS regressions, and we follow this plan throughout. The plan explained that we would estimate treatment effects on a sample of dates ranging from 14 days before to five days after the campaign, which we refer to here as our *restricted sample*. We also pre-registered an intention to explore wider date ranges, given uncertainty about how quickly treatment effects would onset.

Controlling for Differential Trends by Population Size

After the campaign ran, however, we learned an unanticipated feature of the data that necessitated a modification of our analysis: including controls for differential growth rates across counties of different sizes. During the period of our study, county-level growth rates in vaccination counts were very different in counties of different population sizes, leading us to include controls for differential growth rates in counties of different sizes. To illustrate this differential growth, we first estimate a version of equation (1) without including the $Population_i \times Post_t$ term. The results are shown in column 1 of Table S11, where we observe a small point estimate (9.8) that is very imprecisely measured. The 95% confidence interval contains our preferred estimate from the body of the paper, 102.6 (from column 1 of Table 1). We then estimate a version of equation (1) without the $Treat_i \times Post_t$ interaction but including the $Population_i \times Post_t$ term. As in equation (1), the main effect of $Population_i$ is absorbed by county-level effects γ_i. The results are shown column 2 of Table S11. We find a statistically significant and positive coefficient on the $Population_i \times Post_t$ term, implying that a county with 10,000 more residents has 275 more vaccinations in the post period. This increase is entirely independent of our experiment, as the results hold across all counties (column 2) and even within control counties alone (column 3). This suggests that, by not controlling for differential growth in vaccines in counties of different sizes, the specification in column 1 leaves a significant amount of statistical noise uncontrolled for. When we include the $Population_i \times Post_t$ interaction, we obtain the effect of 102.6 reported in column 1 of Table 1.

An additional, minor departure from our pre-registration plan is the following: the plan described omitting the campaign start date (October 14) from our analysis. We replicated our analysis with and without this date and found that the qualitative and quantitative findings of the study were unchanged.

Though our primary analyses deviate from our pre-registered plan, we believe that our revised approach in analyses are all appropriate responses to changes in the research environment that could not be sufficiently anticipated at the time of the pre-registration. We believe, nonetheless, that they are in keeping with the spirit of our pre-registration plan and, under the circumstances, provide the most appropriate tools to assess the causal impact of our advertising campaign on vaccine uptake in the targeted counties.

Examining Different Date Windows and County-level Regressions

As outlined in our pre-registration plan, we estimated treatment effect first on a restricted sample of dates and then moved to wider date ranges. Therefore, exploring different date windows was not a departure from our pre-registration plan. We illustrate these results here to compare the specification with and without population controls under different time windows. The restricted sample of dates is a window from 14 days before to 5 days after the campaign, totaling to 72,815 county-date observations. Estimates of the intent-to-treat effect on this restricted sample are shown in columns 4-5 of Table S11, with column 4 omitting the $Population_i \times Post_t$ interaction and column 5 including it. The results are too imprecisely measured to detect a significant effect in either column, but in the latter the 95% confidence interval contains our preferred estimate from column 1 of Table 1. The final sample we chose to focus on is a wider date range, including dates from one month before the campaign to one month after, which we refer to as our *full sample* (the 151,945 county-date observations). We arrived at this window after exploring the event study presented in the Results section. This event study clearly reveals that the ad campaign affected vaccine counts with a lag, underlining the importance of allowing for a wider date range. The Results section discusses several possible sources for this lag.

We now present an alternative regression design that suggests our estimates are not overly sensitive to either the inclusion of population controls or the time window. In this alternative framework, we create a dataset with one observation per county, estimating the following regression:

$$VaccinesPost_i = \alpha + \beta Treat_i + \gamma VaccinesPre_i + \varepsilon_i \tag{6}$$

where $VaccinesPost_i$ is the number of vaccines in county *i* on a particular date after the campaign (we examine both November 5 and 30) and $VaccinesPre_i$ is the number on a particular date prior to the campaign (we examine both September 30 and 15). In this regression, β captures the intent-to-treat effect by controlling for the level of vaccines in the pre period and estimating the increase in vaccines due to treatment assignment. Note that, as this regression only includes one observation per county, the need to cluster disappears and heteroskedasticity-robust standard errors are appropriate.

The results of this regression are shown in columns 1 and 3 of Table S12. We find a point estimate of 97 in column 1 when we use the narrow date span (Sep. 30 to Nov. 5) and 114 in column 3 when we use the wider date span (Sep. 15 to Nov. 30). Regardless of the date window, the estimates are similar in size to our main ITT estimate of 103. However, these effects are less precisely estimated than the estimates from the county-by-date panel dataset we use for our main analysis. In columns 2

and 4 we also control for the population of county *i*. This mirrors the inclusion of the $Population_i \times Post_t$ interaction in our main panel analysis, allowing for the possibility that vaccine counts increase by more in large counties than in small counties. However, unlike our panel analysis, here these population controls make little difference (the point estimates in columns 2 and 4 are similar to those in columns 1 and 3) because $VaccinesPre_i$ already captures much of the difference in population across counties.

In columns 5-8 of Table S12, we modify regression (6) by omitting $VaccinesPre_i$ from the right-hand side and changing the outcome to $(VaccinesPost_i - VaccinesPre_i)$. In columns 6 and 8 we also include population as a control. In this case, the inclusion of population controls does matter. This is because the left-hand side is a difference and, without controlling for population, nothing in the regression accounts for the dramatic difference in vaccine growth between large and small counties, analogous to how nothing in regression (1) accounts for this differential if the $Population_i \times Post_t$ interaction is omitted. Without controlling for population, the point estimates in columns 5 and 7 of Table S12 are quite different from those in columns 1-4. After controlling for population, the results in columns 6 and 8 are essentially the same as those in columns 1-4, and, again, quite similar to our main estimated effect of 103 vaccines. Finally, as in columns 1-4, the estimates in columns 6-8 do not change drastically as we change the date window.

While this alternative regression framework yields point estimates that are similar to our main results, we prefer the panel approach described in the body of the paper as it yields more precise estimates, using information from all dates rather than just one date before and one date after the campaign.

D: Survey Instrumentation

We contracted with Qualtrics to gather six different 2,400 respondent samples at regular intervals between April 2020 and September 2021 to measure public opinion about COVID-19. Qualtrics conducts Census-targeted sampling to ensure that survey samples closely match the U.S. benchmarks for age, education, gender, and race/ethnicity. The instrument fielded in March/April, as well as August/September, each asked respondents how much confidence they had in several sources "when it comes to advising you on taking the COVID-19 vaccine." In the March/April wave, 422 participants categorized themselves as Republicans and as unvaccinated (i.e., they did not select "I have already

been vaccinated" in response to the question below). In the August/September wave, this number was 387. The exact wording for questions used in the Table 1 analysis appears below.

How much confidence do you have in each of the following when it comes to advising you on taking the COVID-19 vaccine?

	A great deal of confidence (1)	A fair amount (2)	Not too much (3)	No confidence at all (4)	Not Applicable (5)
Donald Trump	o	o	o	o	o
Joe Biden	o	o	o	o	o
Director of the U.S. National Institute of Allergy and Infectious Diseases Dr. Anthony Fauci	o	o	o	o	o
Your personal doctor	o	o	o	o	o
The scientific community	o	o	o	o	o

[March/April 2021 version] ***You may have heard that a COVID-19 vaccine has been authorized for distribution in the United States. If the vaccine were available for you to take today, would you***

- o Definitely get the vaccine
- o Probably get the vaccine
- o Probably NOT get the vaccine
- o Definitely NOT get the vaccine
- o I have already been vaccinated

[August/September 2021 version] ***You likely heard that a COVID 19 vaccine has been authorized for distribution in the United States. How likely are you to get one of the COVID 19 vaccines?***

- o Definitely will get the vaccine
- o Probably will get the vaccine
- o Probably will NOT get the vaccine
- o Definitely will NOT get the vaccine
- o I have already received at least one dose

Generally speaking, do you usually think of yourself as a DEMOCRAT, a REPUBLICAN, an INDEPENDENT, or what?

- o Democrat
- o Republican
- o Independent
- o No preference
- o Other party (specify) ______________________________

Table S1: Regression Results Omitting Counties with Misrecorded Data

	Counties Without Negative Counts				Counties Without Missing Counts			
	ITT Effect		ACR of 1,000 Ads		ITT Effect		ACR of 1,000 Ads	
	(1)	(2)	(3)	(4)	(5)	(6)	(7)	(8)
Treat × Post	114.2*	112.9*			103.3	103.3		
	(83.44)	(83.46)			(80.89)	(80.91)		
Ads × Post			9.014*	8.914*			8.633	8.633
			(6.608)	(6.609)			(6.768)	(6.769)
Pop. × Post	285.5***		273.8***		276.5***		265.6***	
	(20.24)		(23.17)		(18.93)		(21.94)	
County Effects	Yes	Yes	Yes	Yes	Yes	Yes	Yes	Yes
Date Effects	Yes	Yes	Yes	Yes	Yes	Yes	Yes	Yes
Pop. × Post	Yes		Yes		Yes		Yes	
Pop. × Dates		Yes		Yes		Yes		Yes
RI p-value	0.067	0.062	0.067	0.062	0.075	0.075	0.075	0.075
Observations	116163	116163	116163	116163	144144	144144	144144	144144

Columns 1-4 report regression results from the same specifications as in Table 2 but using only counties in which CDC records do not show a decrease in the cumulative vaccination count for any date. Columns 5-8 report results as in Table 2 but using only counties in which CDC records are not missing for any date. ITT refers to intent-to-treat and ACR to average causal response. "***", "**", and "*" indicate significance (from a one-tailed test) at the 0.01, 0.05, and 0.10 levels. Standard errors, reported in parentheses below each estimate, are clustered at the county level. Randomization inference p-values are from a one-tailed test based on 1,000 permutations using the treatment effect – (Treat x Post) in ITT columns and (Ads per 100 x Post) in ACR columns – as the randomization test statistic.

Table S2: Pairwise Correlations Between Measures of Treatment Intensity

	# Ads	# Ads/100 residents	Engage. rate	View rate	Click rate	CPM	% Trump	% College	% White	County Pop.	# Internet
# Ads/100 residents	0.42***										
Engagement rate	-0.16***	-0.05									
View rate	0.01	0.10***	0.71***								
Click rate	0.06**	0.06*	0.00	0.03							
CPM	0.02	0.03	0.61***	0.40***	0.07**						
% Trump	-0.25***	-0.15***	0.18***	-0.05*	-0.07**	-0.08***					
% College	0.24***	0.22***	-0.19***	-0.16***	0.05	-0.02	-0.19***				
% White	-0.16***	-0.01	0.16***	-0.14***	-0.04	-0.07**	0.66***	0.18***			
County Pop	0.94***	0.27***	-0.18***	0.00	0.07**	0.03	-0.27***	0.27***	-0.16***		
# House. w/Internet	0.93***	0.28***	-0.18***	-0.01	0.07**	0.03	-0.26***	0.30***	-0.14***	0.99***	
Frac. House w/Internet	0.26***	0.32***	-0.12***	-0.07**	0.02	-0.08**	-0.04	0.51***	0.26***	0.29***	0.35***

Table displays pairwise correlations between various county-level variables among treatment counties only. In examining statistical significance of these correlation coefficients, we use a two-tailed hypothesis test. These are therefore the only results in the paper where "***", "**", and "*" indicate significance at the 0.01, 0.05, and 0.10 levels under a two-tailed test.

Table S3: IV Regressions with Heterogeneous First Stage

	(1)	(2)
Ads × Post	10.85*	10.87*
	(8.365)	(8.364)
Pop. × Post	253.4***	
	(27.52)	
County Fixed Effects	Yes	Yes
Date Fixed Effects	Yes	Yes
Characteristics × Post Dummies	Yes	Yes
Pop. × Post Dummy	Yes	
Pop. × Date Dummies		Yes
Observations	143816	143816

Results corresponding to those in Table 1 columns 3-4 but replacing the homogenous first stage with a heterogeneous first stage regression. Sample size is smaller than in Table 1 because percent Trump voters and percent of households with internet access are missing for some counties. All regressions include fixed effects at the county and date levels and interactions of the county-characteristics indicators with the Post dummy, as described in Section A of the supplemental materials. "***", "**", and "*" indicate significance (from a one-tailed test) at the 0.01, 0.05, and 0.10 levels. Standard errors, reported in parentheses below each estimate, are clustered at the county level.

Table S4: Vaccine Increase Per County

	Intent-to-Treat Effect		Average Causal Response of 1,000 Ads	
	(1)	(2)	(3)	(4)
Treat × Post	102.6*	101.4*		
	(78.74)	(78.76)		
Ads × Post			8.606*	8.500*
			(6.608)	(6.609)
Pop. × Post	275.4***		264.5***	
	(18.71)		(21.68)	
County Fixed Effects	Yes	Yes	Yes	Yes
Date Fixed Effects	Yes	Yes	Yes	Yes
Pop. × Post Dummy	Yes		Yes	
Pop. × Date Dummies		Yes		Yes
Randomization Inference p-value	0.067	0.065	0.067	0.065
Observations	151945	151945	151945	151945

Full regression estimates corresponding to results from Table 2. All regressions include fixed effects at the county and date levels. Columns 1-2 correspond to regression (1) and columns 3-4 correspond to regression (2). Columns 2 and 4 replaces the $Population_i \times Post_t$ interaction with interactions of county population with (i) dummies for each date within two weeks before to two weeks after the campaign (omitting the date before the campaign started), (ii) a dummy variable for two weeks or more before, and (iii) a dummy variable for two weeks or more after. "***", "**", and "*" indicate significance (from a one-tailed test) at the 0.01, 0.05, and 0.10 levels. Standard errors, reported in parentheses below each estimate, are clustered at the county level. Randomization inference p-values are from a one-tailed test based on 1,000 permutations using the ITT effect (Treat x Post) as the randomization test statistic.

Table S5: Measuring Vaccines in Rates

	Intent-to-Treat Effect		Average Causal Response of 1,000 Ads	
**A.** Unweighted Regression	(1)	(2)	(3)	(4)
Treat × Post	0.570*	0.563*		
	(0.437)	(0.437)		
(Ads per 100) × Post			0.0296*	0.0291*
			(0.0227)	(0.0226)
Pop. × Post		-0.0234**		-0.0270**
		(0.0134)		(0.0139)
Randomization Inference p-value	0.089	0.093	0.089	0.093
**B.** Weighted Regression	(1)	(2)	(3)	(4)
Treat x Post	0.448	0.411		
	(0.458)	(0.454)		
(Ads per 100) x Post			0.0191	0.0173
			(0.0196)	(0.0192)
Pop. x Post		-0.0101**		-0.0113**
		(0.00561)		(0.00590)
Randomization Inference p-value	0.159	0.184	0.159	0.184
County Fixed Effects	Yes	Yes	Yes	Yes
Date Fixed Effects	Yes	Yes	Yes	Yes
Observations	163856	163856	163856	163856

Full regression estimates corresponding to results from Table 4. Estimates come from modifications of regressions (1) and (2) where the dependent variable is the total percent of the county population vaccinated at a given point in time and the treatment intensity is measured as the number of ads a county receives per 100 residents. The number of observations is slightly higher in this table than in our main analysis (163,856 county-date observations rather than 151,945) because, for some observations, the vaccination count is missing on certain dates in the CDC data even though the vaccination rate is recorded. "***", "**", and "*" indicate significance (from a one-tailed test) at the 0.01, 0.05, and 0.10 levels. Standard errors, reported in parentheses below each estimate, are clustered at the county level. Randomization inference p-values are from a one-tailed test based on 1,000 permutations using the ITT effect (Treat x Post) as the randomization test statistic. Panel A reports results from an unweighted regression and panel B shows results where observations are weighted by county population.

Table S6: Vaccine Increase Per County: Heterogeneous Effects and Causal Responses

	W = Below Median % Trump		W = Below Median % College		W = Below Median % White	
	(1)	(2)	(3)	(4)	(5)	(6)
Treat × Post × W	260.0*		-56.76		216.3	
	(154.4)		(158.5)		(152.3)	
Ads × Post × W		17.53		4.496		12.15
		(11.19)		(10.84)		(12.18)
Treat x Post	-27.05		131.1		-4.971	
	(45.95)		(152.6)		(54.66)	
Ads x Post		-4.307		7.168		-0.377
		(7.557)		(8.499)		(9.545)
W × Post	69.90	140.4*	-74.95	-126.7	144.7	212.4***
	(95.13)	(76.04)	(104.6)	(80.92)	(95.52)	(74.47)
Pop. x Post	273.0***	257.8***	274.1***	264.1***	272.1***	258.5***
	(19.10)	(23.13)	(19.27)	(22.91)	(19.19)	(22.79)
County Effects	Yes	Yes	Yes	Yes	Yes	Yes
Date Effects	Yes	Yes	Yes	Yes	Yes	Yes
RI p-value	0.096	0.096	1.00	1.00	.149	.149
Observations	151945	151945	151945	151945	151945	151945

Full regression estimates from regressions in Table 3. Odd columns report results from regression (3) and even columns report an IV version of this regression, where we instrument for $(Ads_i \times Post_t)$ and $(Ads_i \times Post_t \times W_i)$using $(Treat_i \times Post_t \times W_i)$ and $(Treat_i \times Post_t)$. *W* is an indicator for whether the value of a given county-level characteristic is below the median of that characteristic across counties in our sample. This characteristic is the 2016 Trump vote share in columns 1-2, the fraction of county residents with a college degree in columns 3-4, and the fraction of county residents who are white in columns 5-6. "***", "**", and "*" indicate significance (from a two-tailed test) at the 0.01, 0.05, and 0.10 levels. The "Effect at W=1" row displays the sum of the coefficients on $(Treat_i \times Post_t \times W_i)$ and $(Treat_i \times Post_t)$ in odd columns and the sum of $(Ads_i \times Post_t)$ and $(Ads_i \times Post_t \times W_i)$ in even columns. Standard errors, reported in parentheses below each estimate, are clustered at the county level. Randomization inference p-values are from a two-tailed test based on 1,000 permutations using the ITT effect in low-relative-to-high counties $(Treat_i \times Post_t \times W_i)$ as the randomization test statistic.

Table S7: Effects on Vaccine Rates: Heterogeneous Effects and Causal Responses

	W = Below Median % Trump		W = Below Median % College		W = Below Median % White	
	(1)	(2)	(3)	(4)	(5)	(6)
Treat × Post × W	1.765**		-0.848		0.727	
	(0.864)		(0.864)		(0.873)	
APH × Post × W		0.0882**		-0.0404		0.0354
		(0.0440)		(0.0435)		(0.0451)
Treat x Post	-0.334		0.946		0.204	
	(0.492)		(0.747)		(0.596)	
APH x Post		-0.0184		0.0459		0.0110
		(0.0272)		(0.0363)		(0.0321)
W × Post	1.827***	1.854***	-2.237***	-2.254***	0.635	0.660
	(0.582)	(0.577)	(0.597)	(0.593)	(0.587)	(0.582)
Pop. x Post	-0.0591***	-0.0650***	-0.0548***	-0.0587***	-0.0370**	-0.0422***
	(0.0156)	(0.0167)	(0.0167)	(0.0173)	(0.0151)	(0.0160)
County Effects	Yes	Yes	Yes	Yes	Yes	Yes
Date Effects	Yes	Yes	Yes	Yes	Yes	Yes
RI p-value	0.041	0.041	1.00	1.00	0.388	0.388
Observations	163856	163856	163856	163856	163856	163856

Table is analogous to Table S5 but here each regression uses the vaccine rate (the fraction of the county vaccinated) rather than the level as the dependent variable. APH stands for "Ads per 100 residents." Odd columns report results from a regression analogous to (3) and even columns report an IV version of this regression, where we instrument for $(APH \times Post_t)$ and $(APH \times Post_t \times W_i)$using $(Treat_i \times Post_t \times W_i)$ and $(Treat_i \times Post_t)$. *W* is an indicator for whether the value of a given county-level characteristic is below the median of that characteristic across counties in our sample. This characteristic is the 2016 Trump vote share in columns 1-2, the fraction of county residents with a college degree in columns 3-4, and the fraction of county residents who are white in columns 5-6. "***", "**", and "*" indicate significance (from a two-tailed test) at the 0.01, 0.05, and 0.10 levels. The "Effect at W=1" row displays the sum of the coefficients on $(Treat_i \times Post_t \times W_i)$ and $(Treat_i \times Post_t)$ in odd columns and the sum of $(APH \times Post_t)$ and $(APH \times Post_t \times W_i)$ in even columns. Standard errors, reported in parentheses below each estimate, are clustered at the county level. Randomization inference p-values are from a two-tailed test based on 1,000 permutations using the ITT effect in low-relative-to-high counties $(Treat_i \times Post_t \times W_i)$ as the randomization test statistic.

Table S8: Descriptive Statistics for YouTube Viewers

	Total Ads	% View 10 Sec.	% View Full	Cost ($) per 1,000 Ads
		A. Gender		
Female	2,963,754	40.80	12.03	8.69
Male	5,290,982	43.76	12.78	8.96
Unknown	3,318,838	33.46	12.02	7.79
		B. Age		
18 - 24	1,593,423	38.94	11.09	8.69
25 - 34	1,189,531	41.16	12.32	9.25
35 - 44	1,010,042	43.15	13.26	9.16
45 - 54	1,197,026	43.28	13.05	8.83
55 - 64	1,615,427	43.64	12.67	8.60
65+	1,375,389	46.26	12.95	8.69
Unknown	3,592,736	34.23	12.11	7.93
		C. Income		
Top 10%	340,894	42.95	11.50	9.67
11–20%	572,874	44.17	12.24	9.55
21–30%	766,789	43.70	12.51	9.56
31–40%	646,236	43.72	12.31	9.44
41–50%	548,432	44.54	12.79	9.40
Lower 50%	2,720,921	45.24	12.75	9.18
Unknown	5,977,428	35.85	12.21	7.81
D. Parental Status				
Parent	1,449,921	43.95	12.71	9.55
Not a parent	3,987,477	44.26	12.17	9.34
Unknown	6,136,176	36.39	12.42	7.81

Descriptive statistics on ad viewers from Google Ads tools, including the total number of ad impressions, the percentage watching at least 10 seconds or the full ad, and the cost per 1,000 ad impressions (CPM). Panel A displays statistics by gender, panel B by age, panel C by income level, and panel D by parental status. In each panel, “unknown” indicates users for which Google does not know a given characteristic.

Table S9: Where the Ad Placed

	# Outlets	Total Ads	% View 10 Sec.	% View Full	Cost ($) per 1,000 Ads
A. Outlet Type					
Mobile application	10,072	2,292,337	31.11	6.83	7.81
Site	840	94,992	37.00	13.71	6.07
YouTube channel	150,284	7,760,170	44.52	16.33	8.68
B. Outlet Characteristics					
Contains "fox"	235	270,293	49.85	17.81	8.48
Contains "news"	1,479	550,878	49.28	17.50	8.47
C. Top YouTube Channels by Number of Ads Placed					
Fox News		214,920	50.73	18.32	8.41
Forbes Breaking News		67,667	48.74	16.39	8.82
YouTube Movies		34,934	51.67	19.37	9.02
penguinz0		34,158	37.22	10.49	8.39
NFL		33,502	42.45	13.78	8.44
Markiplier		24,844	43.15	13.34	8.75
Inside Edition		23,947	36.70	8.92	8.96
NewsNation Now		23,916	50.28	18.97	8.67
Sky News Australia		23,218	52.14	17.05	8.46
NBC News		22,030	52.41	22.11	8.30
D. Type of Device of Viewer					
TV screens		3,510,664	46.00	16.84	8.15
Tablets		1,555,365	39.19	10.72	8.34
Computers		447,873	37.79	9.35	8.77
Mobile phones		6,059,672	36.99	10.42	8.83

Descriptive statistics for the ad campaign from Google Ads tools. Panel A displays characteristics of the campaign for different types of outlets where the ad placed. Panel B displays characteristics for outlets with "fox" or "news" in the outlet name. Panel C displays characteristics of the top 10 YouTube channels (ranked by the number of impressions) on which the ad placed. Panel D displays the type of electronic device on which the user viewed the ad. The total number of ads in panel A sums to slightly less than 11.6 million because, for some ad impressions, Google Ads tools did not provide details on the outlet type.

Table S10: Standard Errors for Alternative Clustering Levels

	Intent-to-Treat Effect		Average Causal Response of 1,000 Ads	
	(1)	(2)	(3)	(4)
Effect	102.6	101.4	8.606	8.5
Alternative Standard Errors				
Heteroskedasticity-robust	(20.15)***	(18.78)***	(1.69)***	(1.58)***
County Clustering	(78.74)*	(78.76)*	(6.61)*	(6.61)*
State Clustering	(46.65)**	(46.72)**	(3.99)**	(3.99)**
Stratum Clustering	(69.76)*	(69.75)*	(4.51)**	(4.52)**
County and Date Clustering	(77.38)*	(77.94)*	(6.49)*	(6.52)*
State and Date Clustering	(47.61)**	(47.85)**	(3.95)**	(3.98)**
Stratum and Date Clustering	(69.21)*	(68.83)*	(4.35)**	(4.36)**
County Fixed Effects	Yes	Yes	Yes	Yes
Date Fixed Effects	Yes	Yes	Yes	Yes
Pop. × Post Dummy	Yes		Yes	
Pop. × Date Dummies		Yes		Yes

Regression results corresponding to Table 2 with alternative standard error estimates, including clustering at the county level (2,032 counties), state level (43 states), or stratum level (20 strata). Heteroskedasticity-robust standard errors use no clusters. The final three rows of standard errors use two-way clustering at the geographical level and at the data level. County clustering corresponds to the standard error estimates reported in Table 2. "***", "**", and "*" indicate significance (from a one-tailed test) at the 0.01, 0.05, and 0.10 levels.

Table S11: Intent-to-Treat Effect Under Alternative Specifications

	(1)	(2)	(3)	(4)	(5)
Treat × Post	9.758			-1.426	43.37
	(139.6)			(68.07)	(39.88)
Pop. × Post		275.3***	273.1***		131.0***
		(18.71)	(27.56)		(9.936)
County Effects	Yes	Yes	Yes	Yes	Yes
Date Effects	Yes	Yes	Yes	Yes	Yes
Sample	Full	Full	Control	Restricted	Restricted
RI p-value for Treat × Post	0.474	–	–	0.516	0.144
Observations	151945	151945	76266	72815	72815

Regression results. Columns 1-2 use the full sample, column 3 uses only control counties, and columns 4-5 use the restricted sample period (14 days before to five days after the campaign). All regressions include fixed effects at the county and date levels. Column 1 runs a version of regression (1) without the $Population_i \times Post_t$ interaction, whereas columns 2-3 run regression (1) without the $Treat_i \times Post_t$ interaction. Columns 4 repeats the specification of column 1, and column 5 repeats the specification of Table 2 column 1, but on the restricted sample of dates. "***", "**", and "*" indicate significance (from a one-tailed test) at the 0.01, 0.05, and 0.10 levels. Standard errors, reported in parentheses below each estimate, are clustered at the county level. Randomization inference p-values are from a one-tailed test based on 1,000 permutations using the ITT effect (Treat x Post) as the randomization test statistic.

Table S12: Alternative Regressions: One Observation per County

	(1)	(2)	(3)	(4)	(5)	(6)	(7)	(8)
Treat	96.66	98.46	114.0	121.5	17.85	101.3	-91.39	123.1
	(88.76)	(89.26)	(213.2)	(201.0)	(139.4)	(90.18)	(322.8)	(202.9)
Vax Pre	1.048***	1.036***	1.113***	0.953***				
	(0.00363)	(0.0188)	(0.00763)	(0.0696)				
Pop.		65.60		821.8***		250.3***		589.7***
		(88.56)		(346.4)		(19.36)		(41.61)
Pre Date	30Sep	30Sep	15Sep	15Sep	30Sep	30Sep	15Sep	15Sep
Post Date	05Nov	05Nov	30Nov	30Nov	05Nov	05Nov	30Nov	30Nov
RI p-value	0.152	0.147	0.282	0.241	0.444	0.137	0.616	0.241
Obs	1921	1921	1882	1882	1921	1921	1882	1882

Table estimates regression (6) and variants of this regression, with one observation per county. The dates corresponding to Pre and Post in each column are shown in the last two rows. The number of observations is slightly smaller for the wider date window because some counties have missing observations on those dates. "***", "**", and "*" indicate significance (from a one-tailed test) at the 0.01, 0.05, and 0.10 levels. Heteroskedasticity-robust standard errors are reported in parentheses below each estimate. Randomization inference p-values are from a one-tailed test based on 1,000 permutations using the ITT effect (Treat) as the randomization test statistic.

Figure S1: Daily Event Study with Standard Errors Clustered at County and Date Levels

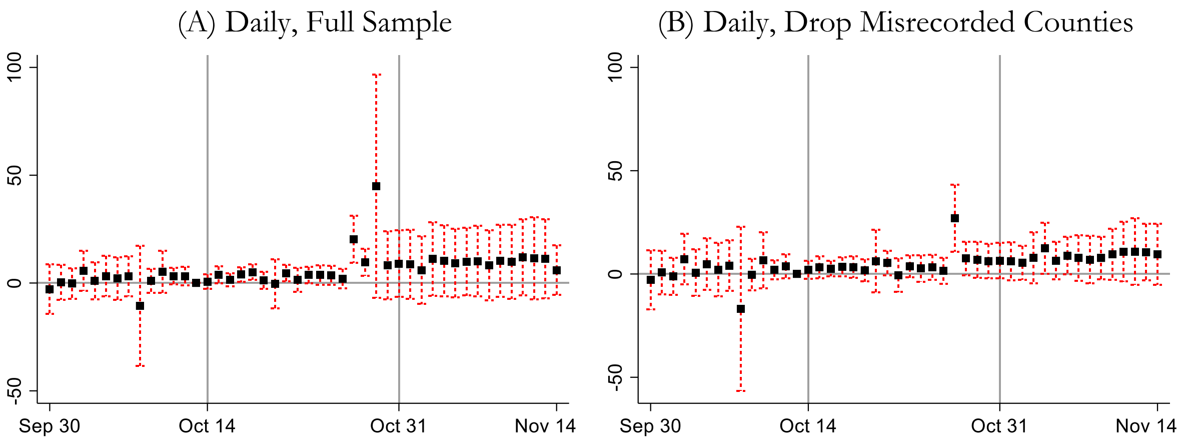

Panels C and D display results from regression (5), the effect on the number of vaccines administered on a given date. Panels on the left use the full sample and those on the right drop counties that ever record a decrease in cumulative vaccine count over time. Red dashed lines representing pointwise 95% confidence intervals are computed under two-way clustering at the county level and date level.

Figure S2: Event Study with Standard Errors Clustered at State and Date Levels

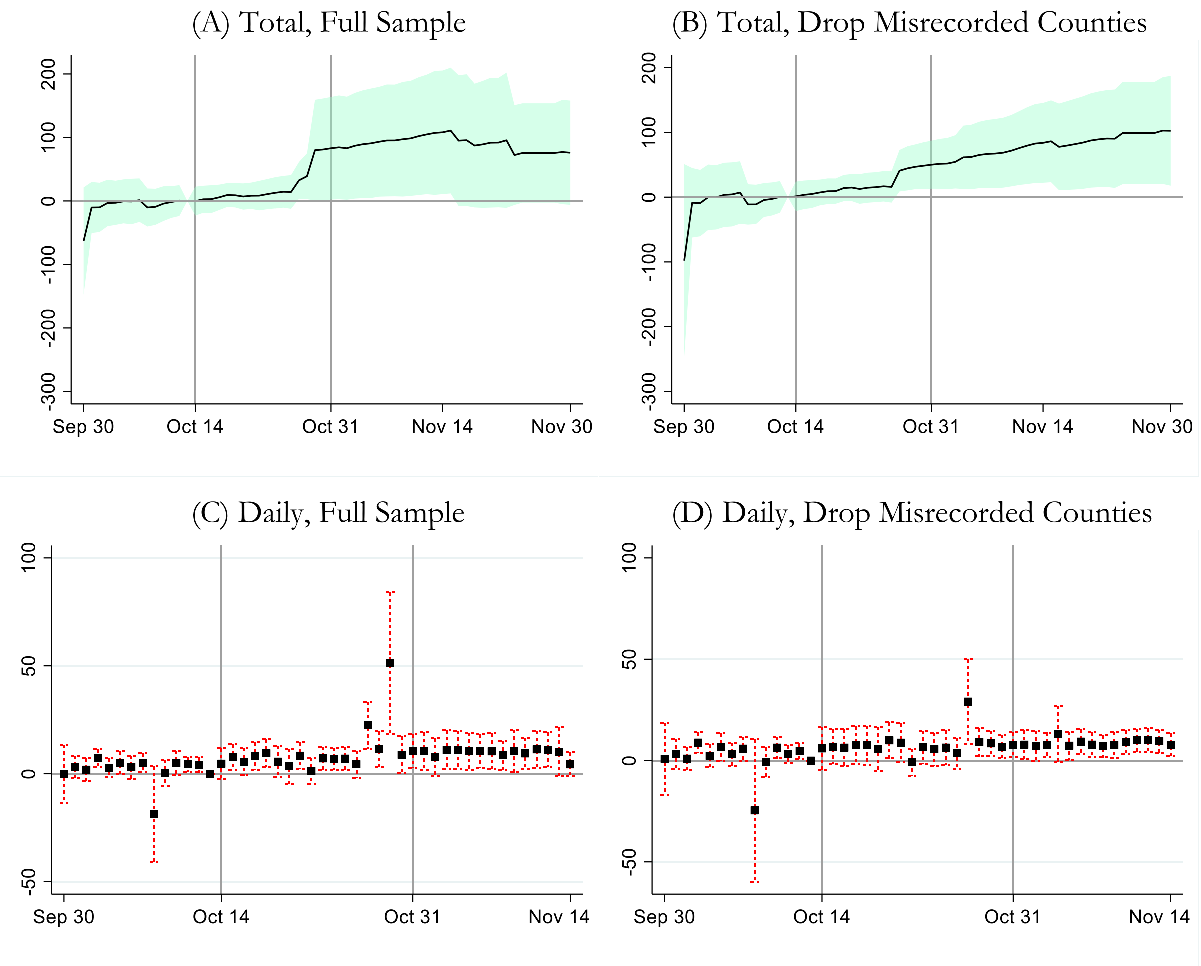

Panels A and B display coefficients from event study regression (4), the effect on the cumulative vaccine count up through a given date. Panels C and D display results from regression (5), the effect on the number of vaccines administered on a given date. Panels on the left use the full sample and those on the right drop counties that ever record a decrease in cumulative vaccine count over time. Shaded region in panels A and B, and red dashed lines in panels C and D, representing pointwise 95% confidence intervals computed under two-way clustering at the state level and date level.

Figure S3: Screenshot Example of a YouTube Segment to which Trump Ad Was Attached

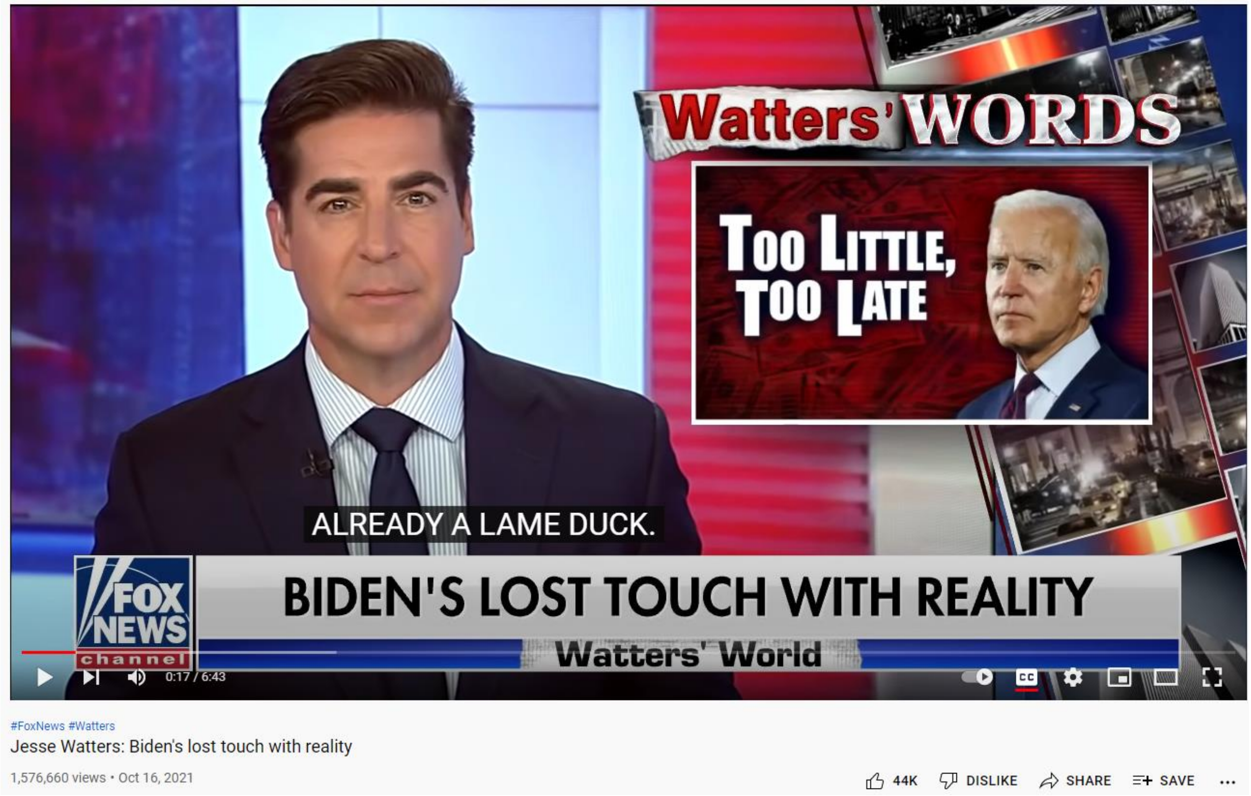

Example of Fox News segment before which our ad was shown (our ad appeared before this segment 2,740 times).
